# Diallelic self‐incompatibility is the main determinant of fertilization patterns in olive orchards

**DOI:** 10.1111/eva.13175

**Published:** 2021-03-05

**Authors:** Roberto Mariotti, Saverio Pandolfi, Isabelle De Cauwer, Pierre Saumitou‐Laprade, Philippe Vernet, Martina Rossi, Federica Baglivo, Luciana Baldoni, Soraya Mousavi

**Affiliations:** ^1^ Institute of Biosciences and Bioresources CNR Perugia Italy; ^2^ CNRS UMR 8198 ‐ Evo‐Eco‐Paleo Univ. Lille Lille France

**Keywords:** incompatibility, olive cultivars, paternity analysis, pollen cloud, pollen donor, stigma test

## Abstract

Self‐incompatibility (SI) in flowering plants potentially represents a major obstacle for sexual reproduction, especially when the number of S‐alleles is low. The situation is extreme in the commercially important olive tree, where in vitro pollination assays suggested the existence of a diallelic SI (DSI) system involving only two groups (G1 and G2). Varieties belonging to the same SI group cannot fertilize each other, such that successful fruit production is predicted to require pollination between varieties of different groups. To test this prediction, we explored the extent to which the DSI system determines fertilization patterns under field conditions. One hundred and seventeen olive cultivars were first genotyped using 10 highly polymorphic dinucleotide Simple Sequence Repeat (SSR) markers to ascertain varietal identity. Cultivars were then phenotyped through controlled pollination tests to assign each of them to one of the two SI groups. We then collected and genotyped 1440 open pollinated embryos from five different orchards constituted of seven local cultivars with known group of incompatibility groups. Embryos genotype information were used: (i) to assign embryos to the most likely pollen donor genotype in the neighbourhood using paternity analysis, and (ii) to compare the composition of the pollen cloud genetic among recipient trees in the five sites. The paternity analysis showed that the DSI system is the main determinant of fertilization success under field open pollination conditions: G1 cultivars sired seeds exclusively on G2 cultivars, and reciprocally. No self‐fertilization events were observed. Our results demonstrate that DSI is a potent force determining pollination success among varieties within olive orchards used for production. They have the potential to improve management practices by guiding the selection of compatible varieties to avoid planting orchards containing sets of varieties with strongly unbalanced SI groups, as these would lead to suboptimal olive production.

## 1 INTRODUCTION

Despite a vast majority of species being hermaphroditic, self‐incompatibility is pervasive among flowering plants (Barrett, 2010). During a given reproductive episode, this mechanism allows avoiding self‐fertilization and inbreeding, but also reduces the number of effective male/female combinations. Understanding how self‐incompatibility shapes the patterns of fertilization is crucial both in wild plant species, where a decrease in the number of compatible partners might threaten long term population persistence (Leducq et al., 2010; Wagenius et al., 2007), and in cultivated crops, where the number of compatible partners can directly impact fruit yield (Muñoz‐Sanz et al., 2020). In self‐incompatible fruit crops, compatible genotypes (often referred to as pollen donors) are frequently spread through fields/orchards, a management practice that is used for instance in apple, pear or apricot (Herrera et al., 2018; Kwon et al., 2015; Sanzol & Robbins, 2008). Despite the crucial impact of the mating system on fertilization patterns, the occurrence/functioning of self‐incompatibility is not known for all crop species.

Cultivated olive (*Olea europaea* L., subsp. *europaea*, var. *europaea*), a wind pollinated species grown for fruit production, blooms profusely and produces pollen in great abundance, but only 2%–3% of more than 500,000 flowers produced by a mature tree usually set fruits (Martin et al., 2005). While it is known that climatic conditions (abundant rain or high temperatures) during the pollination period can contribute to decrease the fruit set (Koubouris et al., 2009; Oteros et al., 2014), the availability of suitable pollen might also be a limiting factor in the field. Indeed, cultivated olive varieties are self‐incompatible and require suitable pollen donors to ensure successful fertilization (Rugini & De Pace, 2016). Self‐incompatibility and incompatibility between genotypes sharing SI specificities potentially represent important reproductive barriers in olive.

It is now well‐established that olive possesses a sporophytic SI system (DSI), characterized by the unusual presence of only two SI specificities (Saumitou‐Laprade, Vernet, Vekemans, Billiard, et al., 2017). Based on robust in vitro stigma tests, each olive genotype can be reliably assigned to one of two SI groups: genotypes belonging to one group are incompatible with each other but compatible with all genotypes of the other group and *vice versa* (but see Breton et al., 2014; Breton et al., 2016; Farinelli et al., 2018). Up to now, this system has been exclusively observed in species within the Oleaceae family, associated with various mating systems: the hermaphrodite cultivated olive (Saumitou‐Laprade, Vernet, Vekemans, Billiard, et al., 2017), the androdioecious *Phillyrea angustifolia* (Billiard et al., 2015; Saumitou‐Laprade et al., 2010) and *Fraxinus ornus* (Vernet et al., 2016), the polygamous *F. excelsior* (Saumitou‐Laprade et al., 2018) and the hermaphrodite *Ligustrum vulgare* (De Cauwer et al., 2020).

A limitation of these data is that most of the empirical evidence obtained so far relies on stigma tests, that is observations of pollen tube trajectories in hand pollinated stigmas. A recent study using paternity analysis in a wild relative, the Saharan wild *Olea europaea* subsp. *laperrinei* (Besnard et al., 2020) confirmed the occurrence of only two SI groups in an experimental population, but the effect of DSI on fruit production remains to be demonstrated directly under field conditions (Farinelli et al., 2018). Obtaining such data is crucial, especially in the context of the current shift to super‐intensive cultivation, which is increasingly based on mono‐varietal olive orchards running the risk of economic failure by lack of consideration of the mating properties of the planted varieties.

In this study, we first characterize the SI phenotypes of 117 of the most commonly used olive varieties in the Mediterranean area, and we provide their multilocus genotype at a set of genetic markers for varietal identification. Based on these data, we determined the extent to which the DSI system is shaping fertilization patterns in field conditions. This knowledge was obtained through paternity analysis of seeds produced in open pollination, after exhaustively genotyping their mother trees and all potential pollen donors in a large 500 m neighbourhood using a set of highly polymorphic genetic markers. Overall, our results provide unequivocal support for the hypothesis that DSI is a potent force under field open pollination conditions and should be taken into account in the design of production orchards.

## 2 MATERIALS AND METHODS

### 2.1 Plant material

#### 2.1.1 Cultivars chosen for correspondence between genotype and SI group

A set of 117 olive cultivars, including elite varieties currently used in the most important olive oil producing countries, such as Arbequina, Koroneiki, Picual and Frantoio, has been phenotyped to determine the incompatibility group through in vitro stigma tests, following the procedure described in Saumitou‐Laprade, Vernet, Vekemans, Billiard, et al. (2017). For 76 cultivars, information on incompatibility groups was extracted from the previous work of Saumitou‐Laprade, Vernet, Vekemans, Billiard, et al., 2017 and Saumitou‐Laprade, Vernet, Vekemans, Castric, et al., 2017) and 41 supplementary cultivars were de novo phenotyped (Table S1). For de novo stigma test experiments, pollen and fresh leaves for DNA extractions were collected from the following olive varietal collections: the World Olive Germplasm Collection of Marrakech (INRA, Morocco), the Zagaria Olive Germplasm Conservation Field (Enna, Italy), the Perugia Collection (Perugia, Italy; Saumitou‐Laprade, Vernet, Vekemans, Billiard, et al., 2017) and the Olive Collection established at CNR‐IBBR (Perugia, Italy). The cross‐compatible cultivars (cvs.) Leccino (belonging to the incompatibility group [G1]) and Dolce Agogia [G2] represented our recipient pair of tester genotypes: their stigmas were used as recipients to phenotype the pollen samples collected from the screened cultivars. Each cultivar was thus phenotyped for SI at the prezygotic stage and genotyped on a set of SSR markers (see below).

#### 2.1.2 Experimental sites description and estimation of varietal composition

Olive orchards are generally constituted of thousands of trees possessing the same genotype and paternity analyses carried out in olive groves need to take into account the clonal origin of individuals, that is several replicas for each genotype, unlike what happens in natural systems in which each potential donor has a genotype different from all the others. Paternity analysis has rarely been used in olive groves due to the technical difficulties of genotyping all potential fathers surrounding the mother trees on which the progenies would be harvested (Baruca Arbeiter et al., 2014; Mookerjee et al., 2005). In the current study, we took advantage of previously gathered information about the identity of varieties in cultivated areas of an Italian olive‐producing region to estimate paternity likelihoods. Given the abundance of olive trees in typical production regions, another difficulty is deciding the relevant scale at which the paternity analysis should be carried out, that is deciding which genotypes should be considered as potential pollen donors. The surface area that one should sample around mother trees to maximize the number of successful pollen donors in the data set will depend on pollen dispersal patterns. Since olive is wind pollinated, one might suspect that pollen can travel over long distances. However, even if pollen can travel over dozen, even hundreds of kilometres carried by the wind (Besnard et al., 2009; Fernández‐Rodríguez et al., 2014; Hernández‐Ceballos et al., 2011; Rojo et al., 2016), paternity analyses usually reveal that long distance effective pollen dispersal is not a ubiquitous characteristic of anemophilous species, including *Olea europaea* subsp. *cuspidata* (Bacles & Ennos, 2008; Burczyk et al., 2002, 2004; Burczyk & Prat, 1997; De Cauwer et al., 2012; Kassa et al., 2018). In this subspecies, over 75% of the pollen dispersal occurred within <200 m around mother trees (Kassa et al., 2018). Assuming pollen dispersal patterns are similar between the two related subspecies, we chose to consider any tree within a radius of 500 m around focal mother plants as a potential pollen donor.

**Figure 1:**
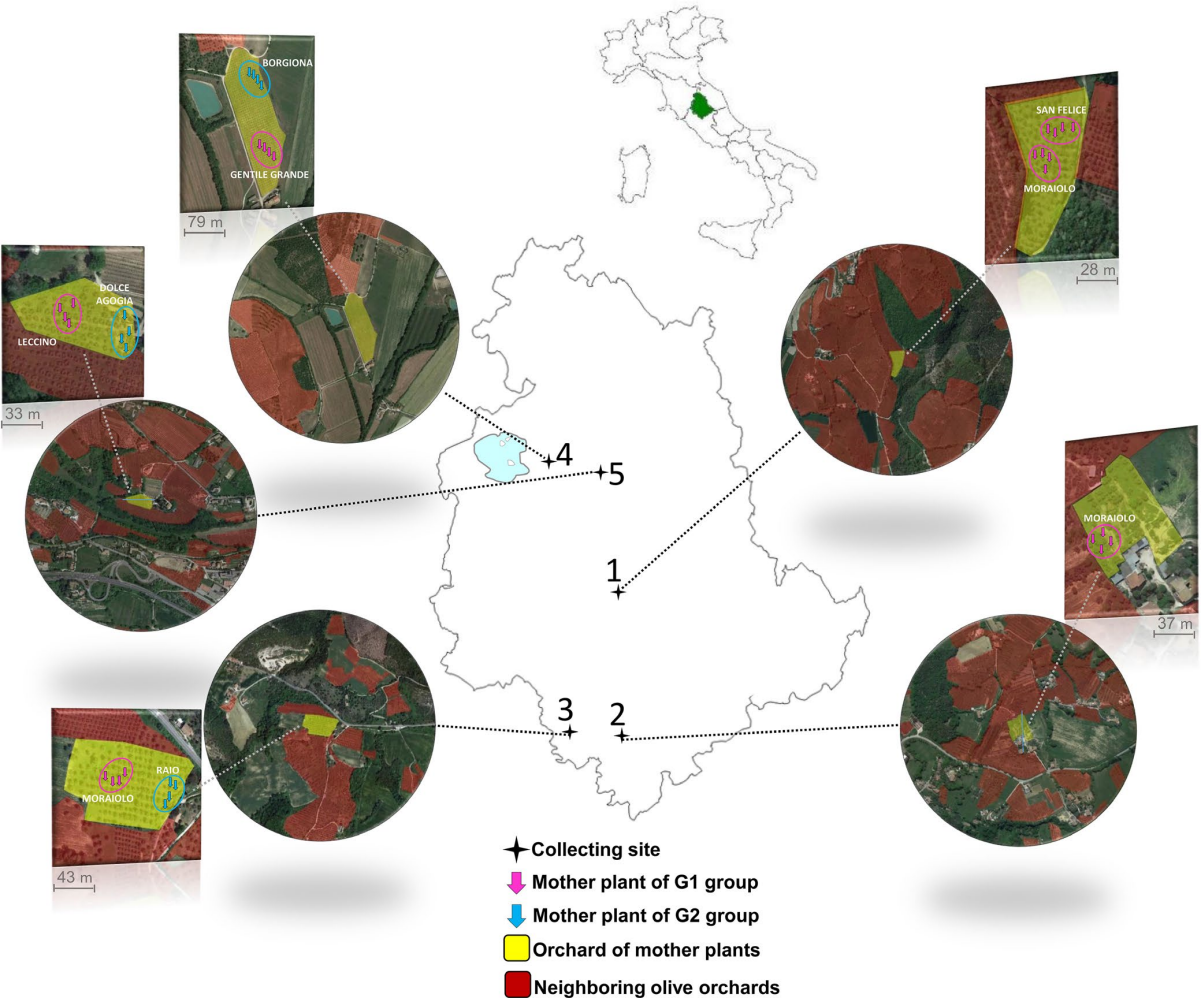
Map of the five study sites in the Umbria region of Italy: Site 1 (Giano dell'Umbria), Site 2 (Narni), Site 3 (Amelia), Site 4 (Corciano) and Site 5 (Perugia). Each circle represents an area of 78.5 hectares (i.e. 500 m around mother trees). The yellow colour highlights the orchard where mother plants were located, while the red colour indicates other olive orchards nearby. Pink (G1) and blue (G2) arrows show the position of each mother tree from which fruits were collected

In order to observe how the DSI system shapes gamete exchanges under field conditions, seven varieties growing in olive orchards of different areas of Umbria region (Italy) were selected for fruit harvesting. Five sites were chosen (Figure 1): Site 1 (Giano dell'Umbria, 42.839 N, 12.573 E), Site 2 (Narni, 42.491 N, 12.493 E), Site 3 (Amelia, 42.539 N, 12.467 E), Site 4 (Corciano, 43.150 N, 12.277 E) and Site 5 (Perugia, 43.097 N, 12.409 E). For each study site, all trees located in an area of 78.5 hectares were considered as potential contributors to the local pollen cloud (Table 1). About 30% of the total surveyed surface (112.06 hectares) corresponded to multiple olive groves and included 24,788 olive trees (Table 1B). The cultivars' occurrence at each site was ascertained by combining the results from previous genotyping work (Baldoni et al., 2003; Mariotti et al., 2009; Pandolfi et al., 2010), specific surveys based on morphological evaluations and interviews with olive producers for each area (Table 1A,B).

**Table 1:** Distribution of the 22 varieties present in the five study sites in Umbria, Italy (Figure 1)

Cultivars	SI group	Sites														
		1			2			3			4			5		
		% within site	% within group	Mother plants	% within site	% within group	Mother plants	% within site	% within group	Mother plants	% within site	% within group	Mother plants	% within site	% within group	Mother plants
A																
Moraiolo	G1	25	29.4	*	65	76.5	*	50	71.4	*	13	18.6		10	15.9	
Gentile Grande	G1										5	7.1	*			
Leccino	G1	15	17.6		10	11.8		10	14.3		20	28.6		20	31.7	*
Frantoio	G1	15	17.6		10	11.8		10	14.3		20	28.6		30	47.6	
San Felice	G1	30	35.3	*												
Villastrada	G1													1	1.6	
Leccio del Corno	G1													2	3.2	
Orbetana	G1										5	7.1				
Pocciolo	G1										5	7.1				
Gentile di Montone	G1										2	2.9				
Pendolino	G2	2	15.4		2	40.0		2	8.0		1	3.4				
Raio	G2							20	80.0	*						
Borgiona	G2										5	17.2	*	2	7.4	
Dolce Agogia	G2	2	15.4								14	48.3		23	85.2	*
Canino	G2				1	20.0		1	4.0							
Bosana	G2	3	23.1								5	17.2				
Maurino	G2	2	15.4		2	40.0		2	8.0							
Nostrale di Rigali	G2	2	15.4								2	6.9				
Coratina	G2													2	7.4	
Itrana	G2										2	6.9			0.0	
Piantone di Mogliano	G2	1	7.7													
Piangente	G2	1	7.7													
Undetermined		2			10			5			1			10		

B	Site 1	Site 2	Site 3	Site 4	Site 5	All Sites
Total surface of olive orchards within site (ha)	41.15	18.87	14.97	11.89	25.18	112.06
Total number of olive trees within site	9115	3275	3319	3431	5648	24,788
Number of [G1] trees	7748	2784	2323	2402	3558	18,815
Total [G1] %	85	85	70	70	63	75.90
Number of G2 trees	1185	164	830	995	1525	4698
Total [G2] %	13	5	25	29	27	19.00
% of undetermined trees	2	10	5	1	10	5.10

Note

A: For each observed cultivar, in column ‘% within site’ is given the percentage of trees from that cultivar within site over the total number of trees present in this site (G1 + G2 + undetermined); in column ‘% within group’ is given the percentage of trees from that cultivar within site over the total number of trees from the same SI group present in this site (G1 or G2). The cultivar of mother plants at each site is indicated as ‘*’. B: For each site are presented: the total surface of olive orchards within site (hectares), the total number of olive trees within site, the number of G1 and G2 trees, as well as their proportion in % over the total number of trees (G1 + G2 + undetermined).

#### 2.1.3 Criteria to select mother plants

In the Umbria region, where the study was conducted, there are about 7.5 million olive trees, corresponding to a few main cultivars. In the National Olive Cultivars Register, Umbria is represented by 11 genotypes, all included in our set of samples, either as mother plants or as possible pollen donors together with dozens of local or minor cultivars, in some cases represented by a few or single trees (Mousavi et al., 2019). Among them, cv. Moraiolo is the most widespread, representing about 35% of total olive trees, whereas cvs. Leccino and Dolce Agogia have a medium diffusion, and cvs. Gentile Grande, Borgiona, San Felice and Raio show a local distribution restricted to a few hectares (Baldoni et al., 2003; Mariotti et al., 2009; Pandolfi et al., 2010).

In order to examine the effects of the site, the genotype and the SI group, seven cultivars grown in five different sites were selected (Table 1A). The site effect was tested by including cv. Moraiolo, a G1 cultivar widely used in Umbria and which was present in three different study sites: 1 (Giano), 2 (Narni) and 3 (Amelia). In order to study the SI group effect, we selected two cultivars belonging to G1 and G2 groups in three sites. In particular, in site 3 (Amelia), we selected the cultivars Moraiolo (G1) and Raio (G2), in site 4 (Corciano) Gentile Grande (G1) and Borgiona (G2) cultivars, and in site 5 (Perugia) the cvs. Leccino (G1) and Dolce Agogia (G2). Finally, in order to assess the genotype effect within site, we selected two (G1) cultivars, Moraiolo and San Felice in site 1 (Giano). In total, we collected fruits from nine different (Genotype × Site) combinations and on seven cultivars (four [G1] and three [G2]) in five different sites (Figure 1). In each site, 160 fruits were collected from four different trees from each cultivar (replicates A, B, C and D), resulting in 36 sampled mothers.

Within each site, the average distance between the four plant replicates (A, B, C and D) of the same genotype was 20 m (Figure 1). Regarding the four sites where two maternal varieties were selected (sites 1, 3, 4 and 5), the average distance between trees belonging to different varieties varied from 24 m (Site 1) to 120 m (Site 4). For each mother tree, healthy fruits were harvested at ripening stage in order to reach at least 10 fully formed embryos from each cardinal direction of the canopy, resulting in a total of 1440 embryos (Table 2 and Figure S1). Pulp and pit were removed from each fruit, seeds were cut‐up with a scalpel and embryos were pulled out and used for DNA extraction.

**Table 2:** Percentage of embryos assigned with relaxed value of 95% confidence to different pollen donors obtained by likelihood‐based paternity tests in the nine cultivar/site combinations (four replicates for each maternal genotype per site, see Table S2)

Sampling sites		Site 1		Site 2		Site 3		Site 1		Site 4		Site 5		Site 3		Site 4		Site 5	
		Pollen recipients																	
		Moraiolo I		Moraiolo II		Moraiolo III		San Felice		Gentile Grande		Leccino		Raio		Borgiona		Dolce Agogia	
Pollen donors	SI Group	G1		G1		G1		G1		G1		G1		G2		G2		G2	
Moraiolo	G1		+		+		+		+		+		+	68.8	+	8.1	+	9.4	+
Gentile Grande	G1		−		−		−		−		+		−		−	75.0	+		−
Leccino	G1		+		+		+		+		+		+	11.9	+	8.8	+	11.9	+
Frantoio	G1		+		+		+		+		+		+	8.1	+	8.1	+	8.8	+
*San Felice*	*G1*		+		−		−		+		−		−		−		−		−
Villastrada	G1		−		−		−		−		−		+		−		−	10.6	+
Leccio del Corno	G1		−		−		−		−		−		+		−		−	10.6	+
*Orbetana*	*G1*		−		−		−		−		+		−		−		+		−
*Pocciolo*	*G1*		−		−		−		−		+		−		−		+		−
*Gentile di Montone*	*G1*		−		−		−		−		+		−		−		+		−
Pendolino	G2	78.1	+	23	+		+	48.8	+	10.0	+		−		+		+		−
Raio	G2		−		−	85.0	+		−		−		−		+		−		−
Borgiona	G2		−		−		−		−	10.0	+	70.0	+		−		+		+
Dolce Agogia	G2		+		−		−	8.1	+	40	+	10.0	+		−		+		+
Canino	G2		−	35	+	8.1	+		−		−		−		+		−		−
Bosana	G2		+		−		−		+	18.8	+		−		−		+		−
Maurino	G2		+		+	6.3	+	10.0	+		−		−		+		−		−
Nostrale di Rigali	G2		+		−		−	13.1	+		+		−		−		+		−
Coratina	G2		−		−		−		−		−	8.1	+		−		−		+
Itrana	G2		−		−		−		−	8.1	+		+		−		+		+
Piantone di Mogliano	G2	0.6	+		−		−	8.1	+		−		−		−		−		−
Piangente	G2	6.3	+		−		−		+		−		−		−		−		−
*Undetermined*		15		42		0.6		11.9		11.9		11.9		11.3		0.0		48.7	
Number of embryos^a^		160		160		160		160		160		160		160		160		160	

Note

Pollen donors (assigned fathers) and pollen recipients (mother plants) are cultivars of olive trees present in Umbria (Table 1A). SI Group: Self‐Incompatibility group for each cultivar, G1 or G2. In grey: within SI group crosses.+/−: presence/absence of trees from the cultivar in the site. In *italics*, the pollen donors never detected among the analysed embryos.

^a^ For each combination (mother‐cultivar/site), paternity assignments are obtained from 160 embryos collected on four trees (40 × 4 = 160) of a single cultivar in a single orchard.

#### 2.1.4 Varietal and embryo genotyping

DNA was extracted from leaf samples of de novo phenotyped trees (41 cultivars), from the 36 mother plants of the field test, as well as from their 1440 embryos, by using the GeneElute Plant Genomic DNA Miniprep Kit (Sigma‐Aldrich) and applying the standard manufacturer's instructions.

These samples were genotyped by using 10 dinucleotide Simple Sequence Repeat (SSR) markers recognized as the most effective for cultivar characterization (Baldoni et al., 2009; El Bakkali et al., 2019; Hosseini‐Mazinani et al., 2014; Mousavi, Mariotti, Bagnoli, et al., 2017; Mousavi, Mariotti, Regni, et al., 2017; Trujillo et al., 2014) and for parentage analyses (Beghe et al., 2017; Kassa et al., 2018; Mookerjee et al., 2005; Seifi et al., 2012). The analysed loci included DCA3‐5‐9‐16‐18 (Sefc et al., 2000), EMO90 (de la Rosa et al., 2002), GAPU71B‐101‐103A (Carriero et al., 2002) and UDO‐043 (Cipriani et al., 2002). PCR amplifications were performed separately in a final reaction volume of 25 μl containing 25 ng of DNA, 1 × PCR buffer, 200 μM of each dNTP, 10 pmol of each forward and reverse primer and 2 U of Q5 High‐Fidelity DNA Polymerase (New England Biolabs), with an initial denaturation at 95°C for 5 min, followed by 40 cycles of 95°C for 30 s, annealing temperature as suggested by authors (50–60°C) for 30 s and 72°C for 25 s, followed by a final elongation at 72°C for 40 min.

PCR products were loaded on an ABI 3130 Genetic Analyzer (Applied Biosystems) using the internal GeneScan 500 LIZ Size Standard (Thermo Fisher Scientific). Fragment sizes were analysed using GeneMapper 3.7 (Applied Biosystems). The profiles of mother plants, four replicates of each genotype from the same olive cultivar, were used as an additional internal controls to select the correct length for each allele during allele calling process.

Varietal identity of all genotyped individuals, including mother plants of the five study sites, was ascertained through the comparison of the SSR data obtained in our study with previously published genotypes (El Bakkali et al., 2019; Mousavi, Mariotti, Bagnoli, et al., 2017; Mousavi, Mariotti, Regni, et al., 2017; Trujillo et al., 2014). A consensus profile was built following the reference allele sizes provided by Baldoni et al. (2009) (Table S1).

GenAlEx 6.501 software (Peakall & Smouse, 2006) was used to calculate the probability of identity (Pi), which provides an estimate of the average probability that two unrelated individuals drawn from the same population will have the same multilocus genotype. Moreover, the same software was utilized to build a genetic distance matrix by using the default parameters for codominant markers (Smouse & Peakall, 1999). The triangular matrix was exported into MEGA X software (Kumar et al., 2018) to construct a neighbour‐joining tree (Saitou & Nei, 1987). A dendrogram was built using the Figtree software 1.4.3 (Rambaut & Drummond, 2012) to allow the visualization of the clustering of individuals and incompatibility phenotypes in the data set.

### 2.2 Paternity analysis

Paternity was determined for the 1440 embryos by using the maximum likelihood‐based method described in Kalinowski et al. (2007) and implemented in CERVUS version 3.0.3 (Marshall et al., 1998). Because of the clonal nature of olive varieties, paternity could not be determined on the basis of individual putative pollen donor trees, but could still be assigned with very high power at the level of individual varieties. Accordingly, we considered all varieties in the 78.5 ha areas surrounding mother trees in a site as potential pollen donors. Using this approach, LOD scores were computed for every embryo—potential paternal variety combination and simulations were used to determine the critical Δ, that is the minimal difference in LOD scores between the two most likely paternal varieties required to assign paternity with 95% confidence. Allele frequencies used in the simulations for each study site were based on the genotypes of locally cultivated varieties weighted by their relative abundances based on orchard surface areas (Table 1A). Within each site, the number of potential paternal genotypes for each incompatibility group varied between 6 (Site 2) and 13 (Site 4). Ten thousand offspring were simulated, allowing for selfing and using the following parameters: the number of candidate fathers was the number of cultivars occurring in each site; the proportion of sampled fathers was estimated based on the surface occupied by determined genotypes in each site (Table 1A). The proportion of ‘typed loci’, ‘mistyped’ and ‘minimum typed loci’ was always set at 0.99, 0.01 and 10, respectively, with a relaxed value of 95% and the strict level at 99%. After simulation runs, paternity analyses were carried out within each site, determining for each embryo the two most likely fathers and deciding whether paternity could be assigned to the most likely father based on the critical Δ obtained from the simulations.

### 2.3 Male gamete heterogeneity among females

The multilocus genotypes of the 36 maternal trees and the 1440 embryos were then used to conduct a gametic analysis of molecular variance within and among mother plants using the TwoGener method, two generational analysis of pollen flow for codominant data (Smouse et al., 2001) implemented in the GenAlex 6.501 software (Peakall & Smouse, 2006). Mother plant/embryo comparisons were used to extract the haploid paternal contributions. A matrix of haploid genetic distances between all pairs of male gametes was built. An AMOVA was then conducted on this gametic distance matrix to partition the overall molecular variance among and within mothers. For all possible mother pairs (i.e. 630 comparisons), the proportion of the molecular variance was estimated using the *φ*_FT_ index. The significance of these pairwise *φ*_FT_ values was assessed using 1000 random permutations of male gametes between mothers.

## 3 RESULTS

### 3.1 Assignment of cultivars to the corresponding incompatibility group and genotype

Based on stigma tests, all 117 varieties could be clearly assigned to either of the two incompatibility groups, in line with Saumitou‐Laprade, Vernet, Vekemans, Billiard, et al. (2017). Specifically, for each variety used as pollen donor, we consistently observed a compatibility reaction on one of the two cultivars used as stigma tester (either Leccino or Dolce Agogia). This compatibility reaction was characterized by several pollen tubes growing and converging through the stigmatic tissue towards the style and successive attrition, such that only one or two tubes reached the style. On the other stigma tester, we observed an incompatibility reaction with the absence of pollen tubes or with the presence of only short pollen tubes growing within the stigma but never reaching the style (Table S1, Figure S2). With no exception, all varieties showed strictly asymmetrical reactions, that is none of them showed either compatibility or incompatibility on both tester lines.

We observed that the two incompatibility groups are equally frequent among Italian (21 G1 and 27 G2; Chi‐square test = 0.386, *df* = 1) and Spanish cultivars (10 G1 and 14 G2; Chi‐square test = 0.234, *df* = 1; Table S1). The neighbour‐joining tree highlighted the presence of synonymous cultivars, which clustered together (Figure S3). In fact, from the 117 phenotyped cultivars, a unique SSR profile was detected for 85 of them. The cases of shared profiles are reported in Table S1. The probability of identity (Pi) was low in our data set (Pi = 7.5. 10^–14^), suggesting that the 10 SSR loci are polymorphic enough to distinguish unrelated individuals. Identical genotypes thus very likely correspond to clonal replicates, originally propagated from the same individual and named differently in various regions or countries. These identical genotypes were always assigned to the same SI group based on the stigma tests. The SSR profiles and SI group of all studied varieties are reported in Table S1. The 36 olive trees selected as mother plants in the paternity analysis below all showed genetic profiles corresponding with their reference variety (seven different genotypes in total). Of these seven varieties, four were phenotyped as (G1), and three as (G2; Table 2).

### 3.2 Paternity analysis

Paternity analysis in each of the five sampling sites allowed assigning paternity to a single variety in 82.91% of cases. The results highlighted the effect of the SI group on fertilization success (Table 2). Among the 1440 progenies analysed, no selfing events were detected, confirming strict self‐incompatibility of the studied cultivars under field conditions. Over the whole data set, 18 different pollen donors were identified. Among the 1188 embryos that were successfully assigned, not a single case of within‐group fertilization was detected (Table 2, Figure S4): the 960 embryos collected from the 24 (G1) mothers were sired by 12 paternal genotypes, all from the (G2) group; whereas the 480 embryos collected from 12 (G2) mothers were sired by six paternal genotypes, all from the (G1) group. Within sites, 20 out of 36 mothers were pollinated mainly by a single cultivar, siring more than 70% of embryos (Table 2).

### 3.3 Heterogeneity of the pollen cloud fertilizing individual trees

The gametic analysis of molecular variance within and among mother plants provides a powerful way to characterize the genetic structure of the pollen cloud. While most of the overall genetic variation in male gametes composition (83%) was found within mother plants, a statistically significant share of the total variation occurred among mothers (*φ*_FT_ = 0.169, *p* < 0.001), indicating that individual trees are pollinated by distinct pollen clouds. Pairwise *φ*_FT_ values computed for all possible mother pairs in the data set are reported in Table S2.

#### 3.3.1 Within‐site/within‐variety pairwise comparisons

Within each of the five selected sites, the four trees sampled for each studied variety allowed us to verify whether replicates of the same genotype located at close vicinity sampled male gametes from the same pollen cloud. Across the 54 within‐site/within‐genotype pairwise comparisons, the average *φ*_FT_ value was low (mean ± SD = 0.002 ± 0.004) and with a single exception were not significantly different from zero (Table S2). Overall, when replicates of the same genotype occurred in the same orchard, they thus tended to sample the same pollen cloud.

#### 3.3.2 Between‐sites/within‐variety pairwise comparisons

Twelve Moraiolo mother plants were sampled in sites 1, 2 and 3 (four replicates per site). Geographic distances between these orchards were as follow: 39.08 km between orchard 1 and 2, 5.75 km between orchard 2 and 3 and 34.67 km between orchards 1 and 3 (Figure 1). All the 48 between‐sites/within‐variety pairwise comparisons were significant, indicating that replicates of the same genotype placed in orchards separated by a few to a few tens of kilometres sampled different pollen clouds (*φ*_FT_ = 0.229 ± 0.066), illustrating that local pollination environments can be different.

#### 3.3.3 Between‐group comparisons

**Figure 2:**
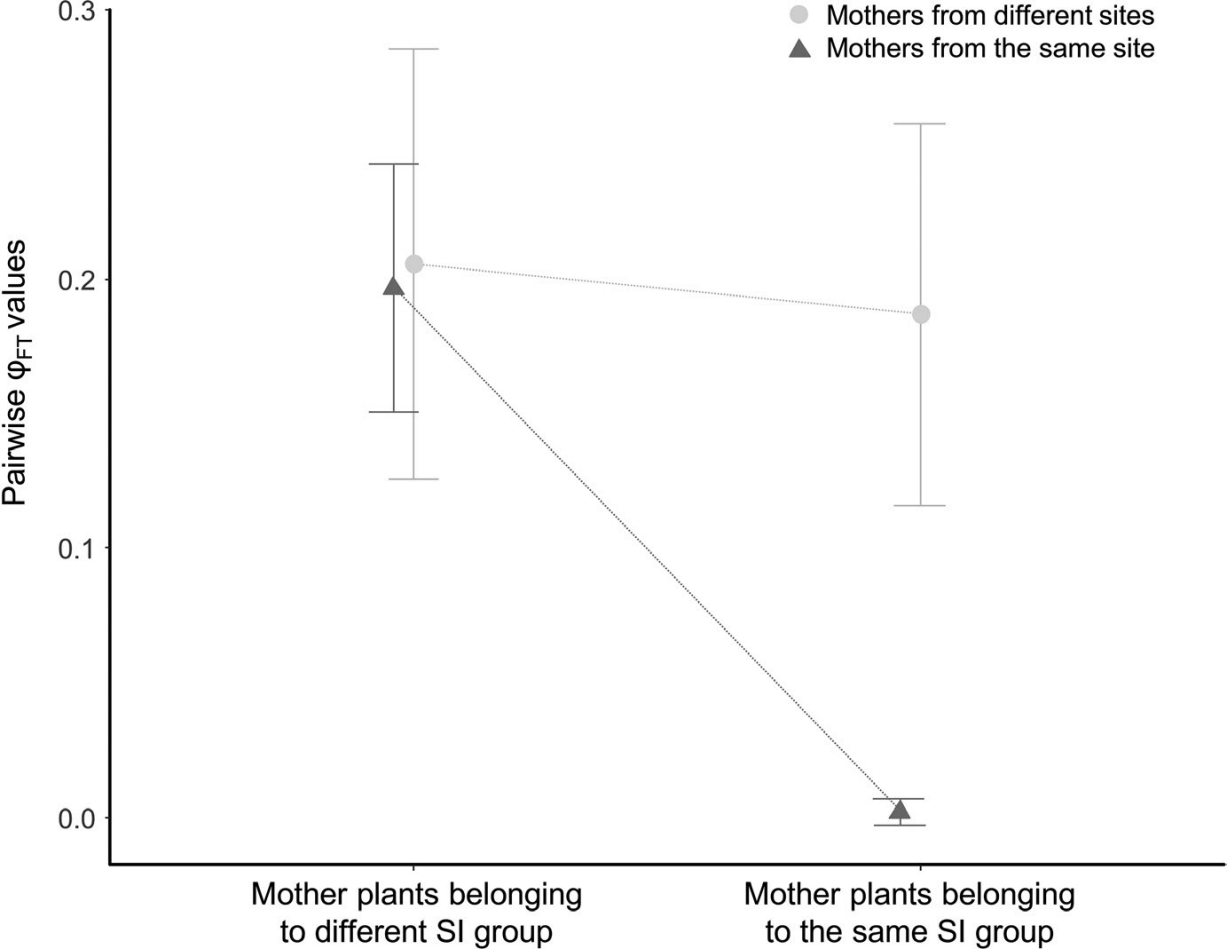
Mean *φ*_FT_ values observed for different types of pairwise comparisons in sites 3, 4 and 5, where mothers from both groups where sampled. The graph summarizes the genetic differences found among and within the collecting sites in relation with incompatibility group

Three study sites included both (G1) and (G2) mothers (site 3: Moraiolo [G1] and Raio [G2]; site 4: Gentile Grande [G1] and Borgiona [G2]; and site 5 Leccino [G1] and Dolce Agogia [G2]), allowing to jointly explore incompatibility group and site effects (Figure 2). Among all possible comparisons involving trees from these three sites, all pairwise *φ*_FT_ values were significant except for within‐site/within‐group comparisons (as described above). Two mother trees located in the same orchard but belonging to different groups sampled male gametes in pollen clouds that were as different as the pollen clouds sampled by two mother trees located in different orchards (Figure 2).

Finally, in one site, two varieties from (G1) were sampled (site 1, with four Moraiolo and four San Felice trees). Within this site, among the 16 comparisons involving the two varieties, 15 showed low but significant pollen cloud genetic differentiation (mean *φ*_FT_ ± SD = 0.034 ± 0.014). This last result highlighted the fact that beyond the strong effects of self‐incompatibility group and among site geographic distance documented above, different genotypes belonging to the same group and located in the same orchard can sample slightly different pollen clouds.

## 4 DISCUSSION

### 4.1 Theoretical predictions and DSI effect in orchards

The discovery of the diallelic self‐incompatibility system in cultivated olive, with the presence of only two inter‐compatible groups, implies that chances of fertile combinations among cultivars are limited to 50% of all possible crosses, if frequencies of each group are equal (Saumitou‐Laprade, Vernet, Vekemans, Billiard, et al., 2017). In areas planted with olive varieties belonging to the same SI group or in mono‐cultivar olive orchards, the limitation in compatible pollen may represent a very strong hindrance to fertilization and fruit production (Guerin et al., 2000). Our study, conducted in five sites in Umbria, exemplifies how strongly the DSI shapes the effective pollen flow in orchards. The results, we obtained in a situation of olive production, are in agreement with the conclusions of a recent study in the Saharan wild *Olea europaea* subsp. *laperrinei* (Besnard et al., 2020). Strict self‐incompatibility appears to be the rule under field conditions for the seven olive cultivars used as mothers in our study and the 37 wild *Olea europaea* subsp. *laperrinei*. A phenomenon, generally thought to be a consequence of environmental factors interfering with the SI reaction or to result from the action of modifier genes (Busch & Schoen, 2008; Levin, 1996), is reported as ‘leaky’ SI. As already discussed in Saumitou‐Laprade, Vernet, Vekemans, Billiard, et al. (2017), it is observed in some olive cultivars like Koroneiki (Marchese et al., 2016) and Casaliva a homonym of Frantoio (Moreno‐Sanz et al., 2020). These cultivars belong to a SI group (Table S1) and present a low rate of selfing.

The main effect of the DSI remains in the distribution of the pollination success detected by the paternity analysis. Among the paternity assignments of 1188 embryos resulting from open pollination of seven different cultivars by 22 different pollen donor varieties, not a single case of within‐group fertilization was detected. This very strict within group incompatibility detected in cultivated olive is also in agreement with the complete absence of progenies produced by crosses within group reported in *Olea europaea* subsp. *laperrinei* (Besnard et al., 2020).

In the olive production areas across the Mediterranean Basin, flower fecundation is expected to be guaranteed by the presence of millions of olive trees; therefore, the availability of compatible pollen should be assured (Aguilera & Ruiz Valenzuela, 2013; Ferri et al., 2008; Pinillos & Cuevas, 2009). Nevertheless, the presence of compatible pollen might seriously decrease in traditional areas of olive cultivation. Indeed, these areas are submitted to cultivar substitution, abandonment of ancient olive trees, plant death after severe winter frost or pathogenic attacks that reduce the number of genotypes present in a region.

At regional scale, within the two most important olive‐producing regions of Spain (Andalucía) and Italy (Apulia), the frequency of the two DSI groups was equilibrate in terms of number of cultivars. In Andalucía, Hojiblanca and Lechin de Sevilla (G1) traditionally grow close to Picual and Manzanilla Cacereña (G2) trees. In Apulia, popular varieties include Ogliarola Barese and Nociara (G1), as well as Coratina and Peranzana (G2). Nevertheless, within the sites we studied in Umbria, while the number of (G1) and (G2) cultivars was similar, the G1 group was strongly overrepresented in terms of number of trees. This bias reaches 85% of (G1) for 5% (G2) and 10% unknown trees in site 2, which may locally result in (G2) pollen limitation.

The unavailability of compatible pollen could represent a serious problem mainly for the new cultivation areas, where olive growing has recently spread away from the traditional cultivation regions, towards other continents or at the opposite hemisphere, without the presence of any other olive or wild relative. The displacement of a few cultivars into new territories, as has occurred in the last 20 years, may result in risky situations because of the potential lack of compatible pollen. In these geographically isolated orchards with intensive mono‐varietal olive plantations, pollination by compatible donors could be compromised (Koubouris et al., 2010; Mehri et al., 2003; Spinardi & Bassi, 2012; Wu et al., 2002). Based on our results, the fruiting failure reported in isolated mono‐varietal orchards (Ayerza & Coates, 2004; Pinillos & Cuevas, 2009) may be related to limitation of compatible pollen, as the presence within orchards of both G1 and G2 cultivars represents a prerequisite for successful fertilization, and hence fruiting.

All these results and observations highlight how the knowledge of the SI group is of utmost importance to optimize pollination success in olive orchard. In order to contribute to this optimization, we provide a first list of 117 cultivars, including clonal cases with different varietal names, typed for SI group using stigma tests and fully genotyped with reliable SSR markers (Baldoni et al., 2009). This set of olive cultivars encompasses a large proportion of the entire variability of cultivated olive (Belaj et al., 2012, 2018; Díez et al., 2012; Mousavi, Mariotti, Regni, et al., 2017) and includes the most important olive cultivars for oil and table olive production.

### 4.2 Fertilization rates among compatible cultivars

Beyond the major effect of the mating system, we observed that recipient trees from different collection sites have been fertilized by genetically differentiated pollen clouds, and to a lesser extent this was also true among varieties sharing a given SI group. Pollen clouds pollinating the same genotype, that is Moraiolo, in three different olive orchards were significantly different. The genetic differentiation was strong and of the same order of magnitude as the differentiation observed between genotypes belonging to two different SI groups. These differences in pollen cloud among sites can be related to the varying contribution of different pollen donors among sites. For instance, on Moraiolo recipients, Pendolino sired 79.4% of embryos in site 1, 23.1% in site 2 and no embryos in site 3. The variable success of this variety was not clearly related to its local abundance within the three sites (15.4%, 40% and 8%, respectively). Explaining such patterns will require additional field experiments, involving more sites, considering the spatial positions of the trees, the main wind direction and the variation in flowering phenology among varieties and among sites.

Even within site, two varieties belonging to the same incompatibility group sampled slightly different pollen clouds, as exemplified in site 1 with the Moraiolo and San Felice recipients. While the level of genetic differentiation detected among mothers belonging to these two varieties was much lower than the levels of pollen cloud differentiation detected among sites or between groups, they were significant and higher than within‐site / within‐variety comparisons. Explaining why two different genotypes belonging to the same SI group do not sample the exact same pollen cloud will also require further studies.

In five out of nine combinations (mother/sites), a single cultivar sired more than 70% of the embryos even if other compatible pollen donors were available locally. Eighty‐five per cent of Moraiolo embryos were pollinated by cv. Raio; the coexistence of these cultivars was found in several ancient olive orchards of that area (Baldoni et al., 2003; Mariotti et al., 2009; Pannelli et al., 2010), highlighting a prominent affinity between these varieties already observed by farmers hundreds of years ago. Pendolino cultivar (G2), previously recognized as a good pollen donor (Solfanelli et al., 2006), was confirmed as a very important pollen donor for (G1) recipient cultivars. It contributed to fertilize four out of six (G1) mother genotypes and was the main pollen donor in one site for cvs. Moraiolo and San Felice, despite its low abundance in that area. The reasons why some compatible cultivars can succeed as pollen donors better than others are not yet established (Montemurro et al., 2019; Selak et al., 2014). Precisely documenting the extent of this intriguing phenomenon and deciphering the factors involved will constitute an interesting next step.

## 5 CONCLUSIONS

The present work confirms the occurrence of only two SI groups within a wide range of genetically verified olive cultivars. It demonstrates how strongly DSI shapes the pollination patterns among cultivars in actual orchards and establishes that the assignment of genotypes to a SI group by in vitro stigma tests in the lab is fully predictive of the complete inability of a cultivar to pollinate another cultivar from the same SI group in the field. Now that the importance of the effect of the DSI on pollination patterns is validated under field conditions, the large‐scale deployment of this approach at an agricultural level will require further analyses to obtain information on the incompatibility group of as much cultivars as possible. This may be facilitated by the recent discovery of STS markers linked to the SI phenotype (Mariotti et al., 2020).

## CONFLICT OF INTEREST

The authors declare no conflicts of interest.

## Supporting information

Fig S1

Fig S2

Fig S3

Fig S4

Table S1

Table S2

## Data Availability

The data that support the findings of this study, which are not already reported in the Supporting information, are available from the corresponding author upon reasonable request.
