# Supplementary figures and images for: Diallelic self‐incompatibility is the main determinant of fertilization patterns in olive orchards
Source: Evol Appl. 2021 Mar 5;14(4):983–95. doi: 10.1111/eva.13175 (PMC8061272; doi:10.1111/eva.13175)

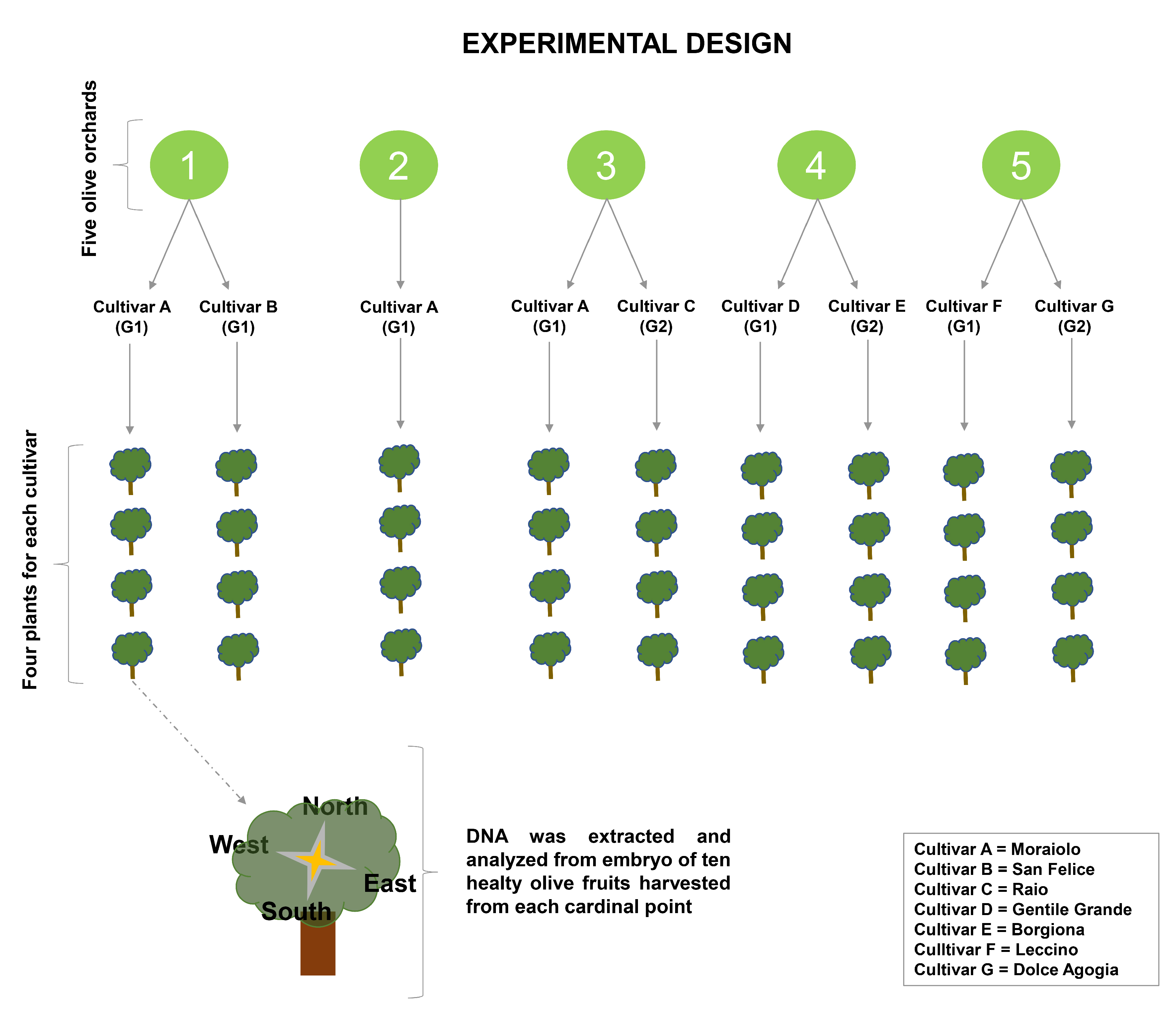

Supplement: Supplementary file 1 — Fig S1 [file EVA-14-983-s006.jpg]

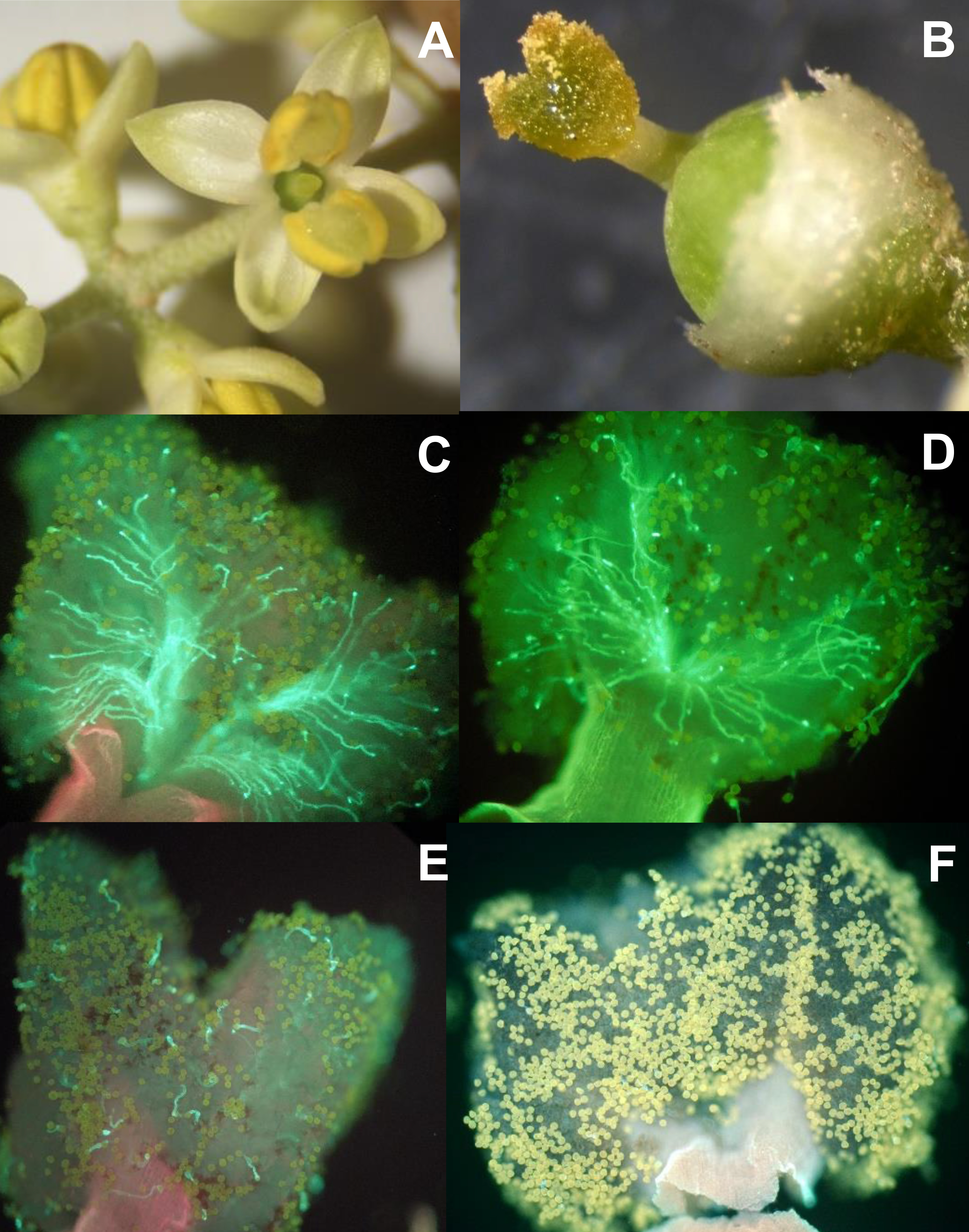

Supplement: Supplementary file 2 — Fig S2 [file EVA-14-983-s002.jpg]

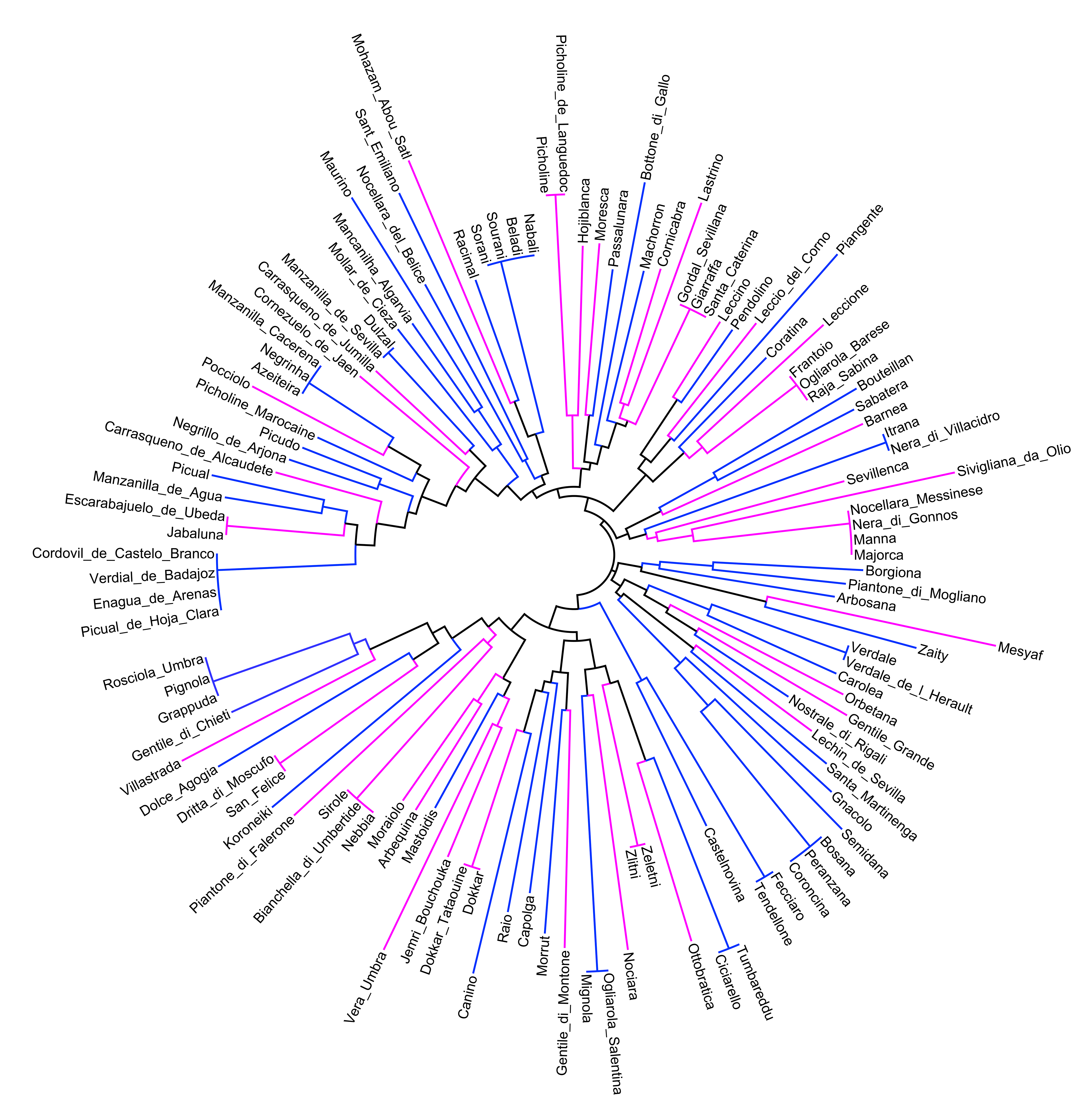

Supplement: Supplementary file 3 — Fig S3 [file EVA-14-983-s001.jpg]

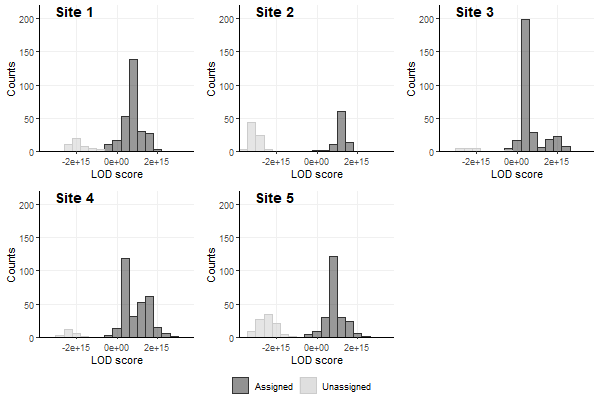

Supplement: Supplementary file 4 — Fig S4 [file EVA-14-983-s005.png]
