# Supplementary material for: Diallelic self‐incompatibility is the main determinant of fertilization patterns in olive orchards
Source: Evol Appl. 2021 Mar 5;14(4):983–95. doi: 10.1111/eva.13175 (PMC8061272; doi:10.1111/eva.13175)
Supplement: Supplementary file 5 — Table S1 [file EVA-14-983-s004.pdf]

Supplementary Table S1. List of 85 olive genotypes corresponding to 117 known cultivars analyzed by stigma test, including those with different names and identical SSR profile. Data on corresponding incompatibility group ([G1] and [G2]), main Country of cultivation, source of plant material (for SSR genotyping and/or stigma test) and consensus allele lengths (bp) at 10 SSR loci are provided. The two incompatibility groups are equally frequent among Italian (21 G1 and 27 G2; Chi^2 test = 0.386, df = 1) and Spanish cultivars (10 G1 and 14 G2; Chi^2 test = 0.234, df = 1)

Cultivar	Cultivars with identical profile	IG^	Main cultivation country	Source of plant material	DCA3	DCA5	DCA9	DCA16	DCA18	EMO90	GAPU71B	GAPU101	GAPU103A	UDO-43
**Arbequina***		G1	Spain	CNR-IBBR[1], UNIPGPerugia-Collection[2]	**231/243**	**202/206**	**184/206**	**124/146**	**169/179**	**188/194**	**124/144**	**183/207**	**150/160**	**176/176**
**Arbosana**		G2	Spain	ZAGARIA[3]	**231/243**	**206/206**	**194/206**	**124/126**	**169/181**	**186/188**	**122/144**	**183/191**	**160/174**	**176/208**
**Azeiteira***	Negrinha, Manzanilla Cacereña	G2	Spain Portugal	UNIPGPerugia-Collection, INRA-CRRA	**239/253**	**206/206**	**162/184**	**124/126**	**173/177**	**188/190**	**122/130**	**199/219**	**136/136**	**174/212**
**Barnea**		G1	Israel	ZAGARIA	**231/231**	**194/206**	**172/194**	**124/146**	**177/179**	**188/190**	**144/148**	**199/201**	**176/188**	**168/176**
**Borgiona#**		G2	Italy	CNR-IBBR	**231/253**	**206/208**	**162/206**	**126/156**	**173/177**	**186/188**	**130/144**	**191/201**	**160/186**	**208/208**
**Bosana**	Peranzana, Coroncina	G2	Italy	CNR-IBBR, ZAGARIA	**243/245**	**200/204**	**172/186**	**124/154**	**179/185**	**188/194**	**128/130**	**193/207**	**174/174**	**176/186**
**Bottone di Gallo***		G2	Italy	ZAGARIA	**239/253**	**194/212**	**166/194**	**174/174**	**177/179**	**188/188**	**122/124**	**195/201**	**150/186**	**172/210**
**Bouteillan***		G2	France	INRA-CRRA(4)	**231/249**	**194/206**	**162/172**	**126/154**	**171/181**	**188/194**	**142/142**	**199/201**	**174/186**	**176/208**
**Canino***		G2	Italy	UNIPGPerugia-Collection, ZAGARIA	**239/239**	**202/206**	**172/184**	**150/150**	**167/179**	**190/190**	**128/144**	**183/199**	**160/174**	**176/186**
**Capolga**		G2	Italy	ZAGARIA	**231/239**	**206/208**	**204/204**	**150/156**	**173/177**	**186/190**	**124/130**	**191/201**	**160/176**	**176/212**
**Carolea***		G2	Italy	UNIPGPerugia-Collection, INRA-CRRA	**231/253**	**194/206**	**162/198**	**126/154**	**179/181**	**188/198**	**122/130**	**193/219**	**136/174**	**174/176**
**Carrasqueño de Alcaudete**		G1	Spain	INRA-CRRA	**239/249**	**206/206**	**184/194**	**126/154**	**171/181**	**188/188**	**122/124**	**199/219**	**136/150**	**174/216**
**Carrasqueño de Jumilla**		G1	Spain	INRA-CRRA	**245/249**	**206/206**	**184/194**	**124/126**	**173/181**	**188/190**	**124/144**	**199/219**	**136/150**	**176/212**
**Castelnovina**		G2	Italy	ZAGARIA	**239/243**	**206/206**	**182/184**	**124/126**	**175/179**	**188/194**	**124/128**	**183/193**	**160/160**	**176/212**
**Coratina***		G2	Italy	UNIPGPerugia-Collection,	**239/243**	**198/206**	**182/194**	**150/174**	**177/181**	**188/194**	**124/144**	**199/219**	**136/162**	**176/198**

Cultivar	Cultivars with identical profile	IG^	Main cultivation country	Source of plant material	DCA3	DCA5	DCA9	DCA16	DCA18	EMO90	GAPU71B	GAPU101	GAPU103A	UDO-43
**Arbequina***		G1	Spain	CNR-IBBR(1), Perugia-Collection(2)	231/243	202/206	184/206	124/146	169/179	188/194	124/144	183/207	150/160	176/176
**Arbosana**		G2	Spain	ZAGARIA(3)	231/243	206/206	194/206	124/126	169/181	186/188	122/144	183/191	160/174	176/208
**Azeiteira***	Negrinha, Manzanilla Cacereña	G2	Spain Portugal	Perugia-Collection, INRA-CRRA	239/253	206/206	162/184	124/126	173/177	188/190	122/130	199/219	136/136	174/212
**Barnea**		G1	Israel	ZAGARIA	231/231	194/206	172/194	124/146	177/179	188/190	144/148	199/201	176/188	168/176
**Borgiona#**		G2	Italy	CNR-IBBR	231/253	206/208	162/206	126/156	173/177	186/188	130/144	191/201	160/186	208/208
**Bosana**	Peranzana, Coroncina	G2	Italy	CNR-IBBR, ZAGARIA	243/245	200/204	172/186	124/154	179/185	188/194	128/130	193/207	174/174	176/186
**Bottone di Gallo***		G2	Italy	ZAGARIA	239/253	194/212	166/194	174/174	177/179	188/188	122/124	195/201	150/186	172/210
**Bouteillan***		G2	France	INRA-CRRA(4)	231/249	194/206	162/172	126/154	171/181	188/194	142/142	199/201	174/186	176/208
**Canino***		G2	Italy	Perugia-Collection, ZAGARIA	239/239	202/206	172/184	150/150	167/179	190/190	128/144	183/199	160/174	176/186
**Capolga**		G2	Italy	ZAGARIA	231/239	206/208	204/204	150/156	173/177	186/190	124/130	191/201	160/176	176/212
**Carolea***		G2	Italy	Perugia-Collection, INRA-CRRA	231/253	194/206	162/198	126/154	179/181	188/198	122/130	193/219	136/174	174/176
**Carrasqueño de Alcaudete**		G1	Spain	INRA-CRRA	239/249	206/206	184/194	126/154	171/181	188/188	122/124	199/219	136/150	174/216
**Carrasqueño de Jumilla**		G1	Spain	INRA-CRRA	245/249	206/206	184/194	124/126	173/181	188/190	124/144	199/219	136/150	176/212
**Castelnovina**		G2	Italy	ZAGARIA	239/243	206/206	182/184	124/126	175/179	188/194	124/128	183/193	160/160	176/212
**Coratina***		G2	Italy	Perugia-Collection, INRA-CRRA, ZAGARIA	239/243	198/206	182/194	150/174	177/181	188/194	124/144	199/219	136/162	176/198

**Cordovil de Castelo Branco***	Verdial de Badajoz, Picual de Hoja Clara, Enagua de Arenas	G2	Spain	INRA-CRRA, Perugia-Collection	**245/253**	**206/206**	**162/184**	**154/176**	**173/181**	**188/188**	**122/124**	**193/219**	**136/136**	**174/212**
**Cornezuelo de Jaen***		G1	Spain	INRA-CRRA, ZAGARIA	**239/253**	**206/210**	**194/204**	**124/126**	**177/181**	**188/190**	**128/144**	**199/219**	**136/150**	**174/216**
**Cornicabra ***		G1	Spain	INRA-CRRA	**239/249**	**206/206**	**184/194**	**124/126**	**173/181**	**188/188**	**124/144**	**193/201**	**186/186**	**174/212**
**Dokkar***	Dokkar Tataouine	G1	Tunisia	INRA-CRRA	**237/239**	**206/216**	**172/196**	**150/150**	**173/181**	**188/190**	**124/130**	**193/199**	**160/174**	**192/212**
**Dolce Agogia*#**		G2	Italy	CNR-IBBR	**231/245**	**194/208**	**172/186**	**124/146**	**171/173**	**186/186**	**124/124**	**191/199**	**160/174**	**172/176**
**Fecciaro***	Tendellone	G2	Italy	ZAGARIA	**243/243**	**200/206**	**176/194**	**126/150**	**175/183**	**188/188**	**124/126**	**193/201**	**160/160**	**172/180**
**Frantoio***	Ogliarola Barese, Raja Sabina	G1	Italy	CNR-IBBR, Perugia-Collection	**237/243**	**198/206**	**182/206**	**150/156**	**177/179**	**188/194**	**124/144**	**183/199**	**162/174**	**176/214**
**Gentile di Chieti***		G2	Italy	Perugia-Collection, INRA-CRRA	**231/243**	**194/212**	**172/172**	**150/154**	**175/177**	**186/190**	**124/130**	**191/199**	**158/174**	**176/214**
**Gentile di Montone**		G1	Italy	CNR-IBBR	**243/253**	**202/202**	**162/186**	**150/156**	**171/173**	**182/190**	**124/130**	**191/197**	**150/160**	**172/176**
**Gentile Grande#**		G1	Italy	CNR-IBBR	**243/253**	**194/206**	**162/172**	**122/174**	**173/185**	**188/194**	**130/144**	**201/207**	**158/186**	**212/212**
**Gnacolo**		G2	Italy	CNR-IBBR	**231/241**	**202/206**	**186/194**	**124/124**	**179/185**	**186/188**	**122/130**	**191/205**	**136/174**	**178/210**
**Gordal Sevillana***	Giarraffa, Santa Caterina	G1	Spain Italy	Perugia-Collection, ZAGARIA	**249/253**	**206/206**	**162/194**	**126/174**	**177/181**	**188/188**	**122/144**	**201/219**	**136/186**	**174/210**
**Grappuda**	Pignola, Rosciola Umbra	G2	Italy	ZAGARIA	**239/245**	**194/204**	**172/186**	**150/154**	**173/185**	**186/190**	**124/130**	**191/199**	**174/174**	**212/214**
**Hojiblanca***		G1	Spain	Perugia-Collection, ZAGARIA	**239/249**	**206/206**	**194/206**	**126/154**	**173/181**	**188/190**	**124/144**	**201/201**	**150/188**	**208/216**
**Itrana***	Nera di Villacidro	G2	Italy	Perugia-Collection, ZAGARIA	**237/247**	**196/204**	**182/194**	**124/126**	**173/181**	**188/188**	**128/144**	**183/201**	**162/188**	**174/176**
**Jabaluna***	Escarabajuelo de Ubeda	G1	Spain	INRA-CRRA	**245/249**	**206/206**	**184/194**	**126/174**	**173/177**	**188/190**	**122/128**	**193/219**	**136/136**	**174/216**
**Jemri Bouchouka***		G1	Tunisia	INRA-CRRA	**231/243**	**194/206**	**172/196**	**124/150**	**177/179**	**190/198**	**124/130**	**193/207**	**150/150**	**176/186**

**Koroneiki***		G2	Greece	Perugia-Collection, ZAGARIA	**239/239**	**194/194**	**182/206**	**146/150**	**173/175**	**188/194**	**124/130**	**193/207**	**150/160**	**172/214**
**Lastrino***		G1	Italy	INRA-CRRA	**245/249**	**206/210**	**194/206**	**126/144**	**173/175**	**188/188**	**122/124**	**183/201**	**186/186**	**210/210**
**Leccino***		G1	Italy	CNR-IBBR, Perugia-Collection	**243/253**	**198/206**	**162/206**	**150/174**	**177/177**	**188/194**	**124/144**	**199/201**	**174/186**	**210/214**
**Leccio del Corno***		G1	Italy	ZAGARIA	**237/253**	**206/206**	**182/206**	**146/156**	**177/179**	**188/194**	**124/144**	**199/201**	**176/192**	**170/212**
**Leccione***		G1	Italy	INRA-CRRA	**243/243**	**206/206**	**162/182**	**146/150**	**177/179**	**188/194**	**124/124**	**191/199**	**162/186**	**176/210**
**Lechin de Sevilla***		G1	Spain	Perugia-Collection, INRA-CRRA	**245/249**	**202/206**	**162/206**	**126/146**	**169/177**	**188/188**	**124/144**	**193/219**	**136/158**	**174/178**
**Machorron***		G2	Spain	INRA-CRRA	**245/249**	**206/206**	**194/208**	**124/174**	**171/181**	**188/188**	**122/130**	**199/201**	**150/186**	**174/212**
**Mancanilha Algarvia**		G2	Spain	INRA-CRRA	**239/253**	**206/206**	**162/206**	**150/174**	**173/177**	**188/194**	**130/144**	**207/221**	**136/150**	**174/212**
**Manzanilla de Agua***		G2	Spain	INRA-CRRA	**239/249**	**206/206**	**194/206**	**126/154**	**173/181**	**188/188**	**122/130**	**193/219**	**136/136**	**174/216**
**Manzanilla de Sevilla***	Dulzal	G2	Spain	Perugia-Collection, INRA-CRRA	**245/253**	**206/206**	**162/206**	**154/174**	**173/181**	**188/190**	**124/144**	**199/219**	**136/150**	**210/214**
**Mastoidis**		G2	Greece	Perugia-Collection	**231/243**	**194/206**	**182/206**	**124/146**	**163/171**	**188/190**	**130/144**	**193/207**	**150/160**	**176/186**
**Maurino***		G2	Italy	Perugia-Collection, ZAGARIA	**237/253**	**206/206**	**206/206**	**150/172**	**177/177**	**188/188**	**144/144**	**185/191**	**162/186**	**174/212**
**Mesyaf***		G1	Morocco	INRA-CRRA	**253/253**	**206/208**	**176/204**	**126/178**	**175/183**	**186/198**	**122/122**	**193/197**	**156/174**	**176/224**
**Mohazam Abou Satl**		G1	Syria	INRA-CRRA	**231/249**	**204/206**	**198/204**	**124/124**	**171/177**	**188/188**	**124/150**	**199/199**	**140/174**	**208/208**
**Mollar de Cieza***		G2	Spain	INRA-CRRA	**239/253**	**206/206**	**162/206**	**124/176**	**173/177**	**188/190**	**122/130**	**199/201**	**150/186**	**174/214**
**Moraiolo*#**		G1	Italy	CNR-IBBR, INRA-CRRA, Perugia-Collection	**231/243**	**206/206**	**184/206**	**146/150**	**179/185**	**188/194**	**124/130**	**193/207**	**150/150**	**176/216**
**Moresca**		G1	Italy	Perugia-Collection, ZAGARIA	**245/249**	**206/206**	**186/194**	**150/174**	**173/181**	**186/188**	**120/144**	**191/201**	**176/188**	**210/214**
**Morrut***		G2	Spain	INRA-CRRA	**243/245**	**198/206**	**186/208**	**150/176**	**173/185**	**190/190**	**128/130**	**183/207**	**150/162**	**174/176**
**Negrillo de Arjona***		G2	Spain	INRA-CRRA	**239/253**	**206/206**	**184/194**	**124/178**	**171/177**	**188/190**	**122/130**	**199/219**	**136/150**	**208/216**

**Nocellara del Belice***		G2	Italy	INRA-CRRA, Perugia-Collection	**243/249**	**206/206**	**162/172**	**150/176**	**173/177**	**188/194**	**124/144**	**199/207**	**150/150**	**172/210**
**Nocellara Messinese***	Nera di Gonnos, Manna, Majorca	G1	Italy	INRA-CRRA, Perugia-Collection	**241/251**	**204/206**	**162/172**	**150/174**	**179/183**	**188/194**	**128/144**	**199/201**	**174/186**	**176/210**
**Nociara***		G1	Italy	INRA-CRRA, ZAGARIA	**237/251**	**198/208**	**166/204**	**150/170**	**169/173**	**188/194**	**124/128**	**199/219**	**176/176**	**176/180**
**Nostrale di Rigali#***		G2	Italy	CNR-IBBR, Perugia-Collection	**243/253**	**194/206**	**162/172**	**126/150**	**173/185**	**188/194**	**122/130**	**199/201**	**174/186**	**174/214**
**Ogliarola Salentina**	Mignola	G2	Italy	ZAGARIA	**243/253**	**198/206**	**172/172**	**146/150**	**159/173**	**194/194**	**124/144**	**183/199**	**160/176**	**178/178**
**Orbetana***		G1	Italy	CNR-IBBR, Perugia-Collection	**243/253**	**194/206**	**162/184**	**126/150**	**173/185**	**188/188**	**124/144**	**193/201**	**190/190**	**174/176**
**Ottobratica***		G1	Italy	INRA-CRRA	**231/239**	**194/198**	**184/202**	**146/154**	**173/177**	**194/194**	**124/124**	**183/207**	**160/174**	**174/176**
**Passalunara***		G2	Italy	Perugia-Collection, ZAGARIA	**243/253**	**206/206**	**186/194**	**150/174**	**171/177**	**186/188**	**122/128**	**191/201**	**174/186**	**174/214**
**Pendolino**		G2	Italy	ZAGARIA	**243/253**	**206/206**	**162/206**	**150/174**	**171/177**	**188/194**	**124/144**	**201/203**	**176/188**	**210/214**
**Piangente**		G2	Italy	ZAGARIA	**243/253**	**206/206**	**162/206**	**146/156**	**177/179**	**186/186**	**124/144**	**183/199**	**164/164**	**172/214**
**Piantone di Falerone**		G1	Italy	CNR-IBBR	**231/251**	**204/208**	**206/206**	**146/150**	**177/179**	**188/194**	**124/130**	**201/207**	**150/184**	**212/212**
**Piantone di Mogliano**		G2	Italy	ZAGARIA	**231/253**	**206/208**	**194/206**	**126/150**	**177/185**	**188/190**	**122/130**	**201/203**	**160/184**	**208/214**
**Picholine Marocaine***		G2	Morocco	Perugia-Collection, ZAGARIA	**239/253**	**206/206**	**194/206**	**154/174**	**173/181**	**188/190**	**122/130**	**199/219**	**136/150**	**174/212**
**Picholine***	Picholine de Languedoc	G1	France	Perugia-Collection, INRA-CRRA	**231/253**	**202/206**	**194/194**	**146/174**	**171/181**	**184/186**	**124/144**	**201/207**	**150/192**	**208/216**
**Picual***		G2	Spain	INRA-CRRA, Perugia-Collection	**239/249**	**206/206**	**184/192**	**126/154**	**171/177**	**188/188**	**122/130**	**193/219**	**136/136**	**208/212**
**Picudo**		G2	Spain	ZAGARIA	**245/253**	**206/206**	**184/194**	**154/176**	**173/177**	**188/190**	**122/124**	**199/219**	**136/150**	**208/216**
**Pocciolo**		G1	Italy	CNR-IBBR, Perugia-Collection	**239/253**	**206/206**	**164/184**	**122/124**	**173/177**	**188/190**	**122/130**	**199/217**	**134/134**	**174/212**

**Racimal***		G2	Spain	INRA-CRRA	245/253	206/206	162/184	124/126	171/177	188/190	122/124	199/199	150/186	208/212
**Raio#**		G2	Italy	CNR-IBBR, ZAGARIA	239/245	200/206	188/204	150/154	171/173	190/198	124/144	193/193	150/160	176/198
**Sabatera**		G2	Spain	INRA-CRRA	231/249	194/206	172/194	126/162	173/181	188/194	124/124	199/201	164/174	176/176
**San Felice#**	Dritta di Moscufo*	G1	Italy	CNR-IBBR, INRA-CRRA	239/239	194/208	172/194	124/172	173/177	186/190	124/130	193/199	150/176	172/176
**Santa Martinenga***		G2	Italy	INRA-CRRA	241/249	202/206	162/186	126/164	171/177	188/198	124/146	193/219	136/160	188/210
**Sant'Emiliano**		G2	Italy	CNR-IBBR	233/243	206/212	194/204	122/156	171/175	188/198	128/144	193/199	150/150	178/178
**Semidana**		G2	Italy	ZAGARIA	245/253	202/204	162/172	124/124	179/179	188/188	130/144	193/219	136/174	176/210
**Sevillenca***		G1	Spain	INRA-CRRA	239/249	206/208	162/182	154/174	177/179	188/188	128/144	183/201	136/160	208/214
**Sirole**	Nebbia, Bianchella di Umbertide	G1	Italy	ZAGARIA	237/239	206/212	182/206	124/146	177/179	186/194	124/130	191/209	150/174	178/216
**Sivigliana da Olio***		G1	Italy	INRA-CRRA	231/253	198/208	162/180	150/176	177/183	188/188	128/128	193/201	160/186	176/176
**Sorani***	Sourani, Beladi, Nabali	G2	Lebanon	INRA-CRRA, Perugia-Collection, CNR-IBBR	245/249	206/206	172/194	124/126	177/177	186/188	124/128	191/199	150/174	210/216
**Tumbareddu**	Ciciarello	G2	Italy	ZAGARIA	231/231	200/206	202/202	124/152	173/175	188/194	124/124	191/207	158/174	176/186
**Vera Umbra**		G1	Italy	ZAGARIA	243/245	194/208	206/206	124/154	173/177	184/184	124/130	193/207	150/150	176/216
**Verdale**	Verdale de l'Herault	G2	France	INRA-CRRA, Perugia-Collection	239/253	194/206	162/172	126/126	179/181	188/194	130/144	207/219	136/150	174/212
**Villastrada**		G1	Italy	CNR-IBBR	237/239	194/198	172/182	146/150	177/187	190/194	124/144	199/199	174/174	214/214
**Zaity***		G2	Syria	INRA-CRRA	249/253	204/206	188/194	126/174	175/183	186/202	122/144	197/207	140/158	176/178
**Zeletni***	Zlitni	G1	Algeria	INRA-CRRA	231/233	198/206	200/206	150/182	171/173	188/194	124/128	191/199	160/178	174/176

^ Incompatibility Group;

* Cultivars already phenotyped in Saumitou-Laprade et al. (2017a and 2017b);

# Mother trees selected in the present study;

(1) CNR-IBBR: CNR - Institute of Biosciences and Bioresources, Div. Perugia, Italy;

(2) Perugia Collection (Perugia, Italy) Saumitou-Laprade et al. (2017a and 2017b);

(3) ZAGARIA: Zagaria Olive Collection (Enna, Italy);

(4) INRA-CRRA: World Olive Germplasm Collection, INRA (Marrakech, Morocco).
