# Supplementary material for: Diallelic self‐incompatibility is the main determinant of fertilization patterns in olive orchards
Source: Evol Appl. 2021 Mar 5;14(4):983–95. doi: 10.1111/eva.13175 (PMC8061272; doi:10.1111/eva.13175)
Supplement: Supplementary file 6 — Table S2 [file EVA-14-983-s003.docx]

**Supplementary Table S2.** Percentages and absolute frequencies of embryos assigned to different pollen donors by paternity analysis. The percentage is calculated on the total number of embryos harvested on the four replicates of a given mother plant genotype assigned to given pollen donor. Plant A, B, C and D indicate the four replicates of 40 seeds assigned for paternity for a given genotype. Pairwise population of φFT values calculated starting on the TwoGener gametic distance matrix: 1,440 seedlings and 36 mother plants were considered using 999 permutations. In bold are reported the cultivars belonging to the DSI group 1.

| **Mother1** | **Mother2** | **φFT** | **#Mother1** | **#Mother2** | **P(rand >= data)** | **No. PW Pm** | **Significance** |
| --- | --- | --- | --- | --- | --- | --- | --- |
| **MORAIOLO_S1_A** | **MORAIOLO_S1_B** | 0.000 | 40 | 40 | 0.417 | 999 | NS |
| **MORAIOLO_S1_A** | **MORAIOLO_S1_C** | 0.000 | 40 | 40 | 0.420 | 999 | NS |
| **MORAIOLO_S1_B** | **MORAIOLO_S1_C** | 0.000 | 40 | 40 | 0.431 | 999 | NS |
| **MORAIOLO_S1_A** | **MORAIOLO_S1_D** | 0.000 | 40 | 40 | 0.416 | 999 | NS |
| **MORAIOLO_S1_B** | **MORAIOLO_S1_D** | 0.000 | 40 | 40 | 0.415 | 999 | NS |
| **MORAIOLO_S1_C** | **MORAIOLO_S1_D** | 0.000 | 40 | 40 | 0.436 | 999 | NS |
| **MORAIOLO_S1_A** | **SAN FELICE_S1_A** | 0.019 | 40 | 40 | 0.034 | 999 | Significant |
| **MORAIOLO_S1_B** | **SAN FELICE_S1_A** | 0.021 | 40 | 40 | 0.023 | 999 | Significant |
| **MORAIOLO_S1_C** | **SAN FELICE_S1_A** | 0.030 | 40 | 40 | 0.009 | 999 | Significant |
| **MORAIOLO_S1_D** | **SAN FELICE_S1_A** | 0.041 | 40 | 40 | 0.003 | 999 | Significant |
| **MORAIOLO_S1_A** | **SAN FELICE_S1_B** | 0.047 | 40 | 40 | 0.002 | 999 | Significant |
| **MORAIOLO_S1_B** | **SAN FELICE_S1_B** | 0.047 | 40 | 40 | 0.002 | 999 | Significant |
| **MORAIOLO_S1_C** | **SAN FELICE_S1_B** | 0.047 | 40 | 40 | 0.001 | 999 | Significant |
| **MORAIOLO_S1_D** | **SAN FELICE_S1_B** | 0.060 | 40 | 40 | 0.002 | 999 | Significant |
| **SAN FELICE_S1_A** | **SAN FELICE_S1_B** | 0.001 | 40 | 40 | 0.387 | 999 | NS |
| **MORAIOLO_S1_A** | **SAN FELICE_S1_C** | 0.015 | 40 | 40 | 0.055 | 999 | NS |
| **MORAIOLO_S1_B** | **SAN FELICE_S1_C** | 0.016 | 40 | 40 | 0.050 | 999 | Significant |
| **MORAIOLO_S1_C** | **SAN FELICE_S1_C** | 0.019 | 40 | 40 | 0.043 | 999 | Significant |
| **MORAIOLO_S1_D** | **SAN FELICE_S1_C** | 0.025 | 40 | 40 | 0.012 | 999 | Significant |
| **SAN FELICE_S1_A** | **SAN FELICE_S1_C** | 0.000 | 40 | 40 | 0.434 | 999 | NS |
| **SAN FELICE_S1_B** | **SAN FELICE_S1_C** | 0.000 | 40 | 40 | 0.421 | 999 | NS |
| **MORAIOLO_S1_A** | **SAN FELICE_S1_D** | 0.030 | 40 | 40 | 0.009 | 999 | Significant |
| **MORAIOLO_S1_B** | **SAN FELICE_S1_D** | 0.040 | 40 | 40 | 0.001 | 999 | Significant |
| **MORAIOLO_S1_C** | **SAN FELICE_S1_D** | 0.036 | 40 | 40 | 0.003 | 999 | Significant |
| **MORAIOLO_S1_D** | **SAN FELICE_S1_D** | 0.046 | 40 | 40 | 0.001 | 999 | Significant |
| **SAN FELICE_S1_A** | **SAN FELICE_S1_D** | 0.008 | 40 | 40 | 0.137 | 999 | NS |
| **SAN FELICE_S1_B** | **SAN FELICE_S1_D** | 0.010 | 40 | 40 | 0.127 | 999 | NS |
| **SAN FELICE_S1_C** | **SAN FELICE_S1_D** | 0.004 | 40 | 40 | 0.262 | 999 | NS |
| **MORAIOLO_S1_A** | **MORAIOLO_S2_A** | 0.145 | 40 | 40 | 0.001 | 999 | Significant |
| **MORAIOLO_S1_B** | **MORAIOLO_S2_A** | 0.140 | 40 | 40 | 0.001 | 999 | Significant |
| **MORAIOLO_S1_C** | **MORAIOLO_S2_A** | 0.147 | 40 | 40 | 0.001 | 999 | Significant |
| **MORAIOLO_S1_D** | **MORAIOLO_S2_A** | 0.151 | 40 | 40 | 0.001 | 999 | Significant |
| **SAN FELICE_S1_A** | **MORAIOLO_S2_A** | 0.108 | 40 | 40 | 0.001 | 999 | Significant |
| **SAN FELICE_S1_B** | **MORAIOLO_S2_A** | 0.087 | 40 | 40 | 0.001 | 999 | Significant |
| **SAN FELICE_S1_C** | **MORAIOLO_S2_A** | 0.109 | 40 | 40 | 0.001 | 999 | Significant |
| **SAN FELICE_S1_D** | **MORAIOLO_S2_A** | 0.112 | 40 | 40 | 0.001 | 999 | Significant |
| **MORAIOLO_S1_A** | **MORAIOLO_S2_B** | 0.152 | 40 | 40 | 0.001 | 999 | Significant |
| **MORAIOLO_S1_B** | **MORAIOLO_S2_B** | 0.140 | 40 | 40 | 0.001 | 999 | Significant |
| **MORAIOLO_S1_C** | **MORAIOLO_S2_B** | 0.154 | 40 | 40 | 0.001 | 999 | Significant |
| **MORAIOLO_S1_D** | **MORAIOLO_S2_B** | 0.153 | 40 | 40 | 0.001 | 999 | Significant |
| **SAN FELICE_S1_A** | **MORAIOLO_S2_B** | 0.113 | 40 | 40 | 0.001 | 999 | Significant |
| **SAN FELICE_S1_B** | **MORAIOLO_S2_B** | 0.100 | 40 | 40 | 0.001 | 999 | Significant |
| **SAN FELICE_S1_C** | **MORAIOLO_S2_B** | 0.115 | 40 | 40 | 0.001 | 999 | Significant |
| **SAN FELICE_S1_D** | **MORAIOLO_S2_B** | 0.121 | 40 | 40 | 0.001 | 999 | Significant |
| **MORAIOLO_S2_A** | **MORAIOLO_S2_B** | 0.000 | 40 | 40 | 0.392 | 999 | NS |
| **MORAIOLO_S1_A** | **MORAIOLO_S2_C** | 0.189 | 40 | 40 | 0.001 | 999 | Significant |
| **MORAIOLO_S1_B** | **MORAIOLO_S2_C** | 0.176 | 40 | 40 | 0.001 | 999 | Significant |
| **MORAIOLO_S1_C** | **MORAIOLO_S2_C** | 0.192 | 40 | 40 | 0.001 | 999 | Significant |
| **MORAIOLO_S1_D** | **MORAIOLO_S2_C** | 0.191 | 40 | 40 | 0.001 | 999 | Significant |
| **SAN FELICE_S1_A** | **MORAIOLO_S2_C** | 0.143 | 40 | 40 | 0.001 | 999 | Significant |
| **SAN FELICE_S1_B** | **MORAIOLO_S2_C** | 0.129 | 40 | 40 | 0.001 | 999 | Significant |
| **SAN FELICE_S1_C** | **MORAIOLO_S2_C** | 0.146 | 40 | 40 | 0.001 | 999 | Significant |
| **SAN FELICE_S1_D** | **MORAIOLO_S2_C** | 0.152 | 40 | 40 | 0.001 | 999 | Significant |
| **MORAIOLO_S2_A** | **MORAIOLO_S2_C** | 0.000 | 40 | 40 | 0.408 | 999 | NS |
| **MORAIOLO_S2_B** | **MORAIOLO_S2_C** | 0.000 | 40 | 40 | 0.416 | 999 | NS |
| **MORAIOLO_S1_A** | **MORAIOLO_S2_D** | 0.189 | 40 | 40 | 0.001 | 999 | Significant |
| **MORAIOLO_S1_B** | **MORAIOLO_S2_D** | 0.174 | 40 | 40 | 0.001 | 999 | Significant |
| **MORAIOLO_S1_C** | **MORAIOLO_S2_D** | 0.189 | 40 | 40 | 0.001 | 999 | Significant |
| **MORAIOLO_S1_D** | **MORAIOLO_S2_D** | 0.189 | 40 | 40 | 0.001 | 999 | Significant |
| **SAN FELICE_S1_A** | **MORAIOLO_S2_D** | 0.140 | 40 | 40 | 0.001 | 999 | Significant |
| **SAN FELICE_S1_B** | **MORAIOLO_S2_D** | 0.123 | 40 | 40 | 0.001 | 999 | Significant |
| **SAN FELICE_S1_C** | **MORAIOLO_S2_D** | 0.143 | 40 | 40 | 0.001 | 999 | Significant |
| **SAN FELICE_S1_D** | **MORAIOLO_S2_D** | 0.152 | 40 | 40 | 0.001 | 999 | Significant |
| **MORAIOLO_S2_A** | **MORAIOLO_S2_D** | 0.000 | 40 | 40 | 0.384 | 999 | NS |
| **MORAIOLO_S2_B** | **MORAIOLO_S2_D** | 0.000 | 40 | 40 | 0.434 | 999 | NS |
| **MORAIOLO_S2_C** | **MORAIOLO_S2_D** | 0.000 | 40 | 40 | 0.393 | 999 | NS |
| **MORAIOLO_S1_A** | **MORAIOLO_S3_A** | 0.314 | 40 | 40 | 0.001 | 999 | Significant |
| **MORAIOLO_S1_B** | **MORAIOLO_S3_A** | 0.317 | 40 | 40 | 0.001 | 999 | Significant |
| **MORAIOLO_S1_C** | **MORAIOLO_S3_A** | 0.314 | 40 | 40 | 0.001 | 999 | Significant |
| **MORAIOLO_S1_D** | **MORAIOLO_S3_A** | 0.324 | 40 | 40 | 0.001 | 999 | Significant |
| **SAN FELICE_S1_A** | **MORAIOLO_S3_A** | 0.259 | 40 | 40 | 0.001 | 999 | Significant |
| **SAN FELICE_S1_B** | **MORAIOLO_S3_A** | 0.232 | 40 | 40 | 0.001 | 999 | Significant |
| **SAN FELICE_S1_C** | **MORAIOLO_S3_A** | 0.260 | 40 | 40 | 0.001 | 999 | Significant |
| **SAN FELICE_S1_D** | **MORAIOLO_S3_A** | 0.270 | 40 | 40 | 0.001 | 999 | Significant |
| **MORAIOLO_S2_A** | **MORAIOLO_S3_A** | 0.195 | 40 | 40 | 0.001 | 999 | Significant |
| **MORAIOLO_S2_B** | **MORAIOLO_S3_A** | 0.208 | 40 | 40 | 0.001 | 999 | Significant |
| **MORAIOLO_S2_C** | **MORAIOLO_S3_A** | 0.217 | 40 | 40 | 0.001 | 999 | Significant |
| **MORAIOLO_S2_D** | **MORAIOLO_S3_A** | 0.203 | 40 | 40 | 0.001 | 999 | Significant |
| **MORAIOLO_S1_A** | **MORAIOLO_S3_B** | 0.291 | 40 | 40 | 0.001 | 999 | Significant |
| **MORAIOLO_S1_B** | **MORAIOLO_S3_B** | 0.297 | 40 | 40 | 0.001 | 999 | Significant |
| **MORAIOLO_S1_C** | **MORAIOLO_S3_B** | 0.292 | 40 | 40 | 0.001 | 999 | Significant |
| **MORAIOLO_S1_D** | **MORAIOLO_S3_B** | 0.305 | 40 | 40 | 0.001 | 999 | Significant |
| **SAN FELICE_S1_A** | **MORAIOLO_S3_B** | 0.239 | 40 | 40 | 0.001 | 999 | Significant |
| **SAN FELICE_S1_B** | **MORAIOLO_S3_B** | 0.206 | 40 | 40 | 0.001 | 999 | Significant |
| **SAN FELICE_S1_C** | **MORAIOLO_S3_B** | 0.239 | 40 | 40 | 0.001 | 999 | Significant |
| **SAN FELICE_S1_D** | **MORAIOLO_S3_B** | 0.247 | 40 | 40 | 0.001 | 999 | Significant |
| **MORAIOLO_S2_A** | **MORAIOLO_S3_B** | 0.183 | 40 | 40 | 0.001 | 999 | Significant |
| **MORAIOLO_S2_B** | **MORAIOLO_S3_B** | 0.198 | 40 | 40 | 0.001 | 999 | Significant |
| **MORAIOLO_S2_C** | **MORAIOLO_S3_B** | 0.209 | 40 | 40 | 0.001 | 999 | Significant |
| **MORAIOLO_S2_D** | **MORAIOLO_S3_B** | 0.199 | 40 | 40 | 0.001 | 999 | Significant |
| **MORAIOLO_S3_A** | **MORAIOLO_S3_B** | 0.000 | 40 | 40 | 0.449 | 999 | NS |
| **MORAIOLO_S1_A** | **MORAIOLO_S3_C** | 0.331 | 40 | 40 | 0.001 | 999 | Significant |
| **MORAIOLO_S1_B** | **MORAIOLO_S3_C** | 0.334 | 40 | 40 | 0.001 | 999 | Significant |
| **MORAIOLO_S1_C** | **MORAIOLO_S3_C** | 0.331 | 40 | 40 | 0.001 | 999 | Significant |
| **MORAIOLO_S1_D** | **MORAIOLO_S3_C** | 0.339 | 40 | 40 | 0.001 | 999 | Significant |
| **SAN FELICE_S1_A** | **MORAIOLO_S3_C** | 0.279 | 40 | 40 | 0.001 | 999 | Significant |
| **SAN FELICE_S1_B** | **MORAIOLO_S3_C** | 0.248 | 40 | 40 | 0.001 | 999 | Significant |
| **SAN FELICE_S1_C** | **MORAIOLO_S3_C** | 0.281 | 40 | 40 | 0.001 | 999 | Significant |
| **SAN FELICE_S1_D** | **MORAIOLO_S3_C** | 0.283 | 40 | 40 | 0.001 | 999 | Significant |
| **MORAIOLO_S2_A** | **MORAIOLO_S3_C** | 0.204 | 40 | 40 | 0.001 | 999 | Significant |
| **MORAIOLO_S2_B** | **MORAIOLO_S3_C** | 0.218 | 40 | 40 | 0.001 | 999 | Significant |
| **MORAIOLO_S2_C** | **MORAIOLO_S3_C** | 0.224 | 40 | 40 | 0.001 | 999 | Significant |
| **MORAIOLO_S2_D** | **MORAIOLO_S3_C** | 0.212 | 40 | 40 | 0.001 | 999 | Significant |
| **MORAIOLO_S3_A** | **MORAIOLO_S3_C** | 0.000 | 40 | 40 | 0.429 | 999 | NS |
| **MORAIOLO_S3_B** | **MORAIOLO_S3_C** | 0.003 | 40 | 40 | 0.313 | 999 | NS |
| **MORAIOLO_S1_A** | **MORAIOLO_S3_D** | 0.311 | 40 | 40 | 0.001 | 999 | Significant |
| **MORAIOLO_S1_B** | **MORAIOLO_S3_D** | 0.314 | 40 | 40 | 0.001 | 999 | Significant |
| **MORAIOLO_S1_C** | **MORAIOLO_S3_D** | 0.311 | 40 | 40 | 0.001 | 999 | Significant |
| **MORAIOLO_S1_D** | **MORAIOLO_S3_D** | 0.322 | 40 | 40 | 0.001 | 999 | Significant |
| **SAN FELICE_S1_A** | **MORAIOLO_S3_D** | 0.256 | 40 | 40 | 0.001 | 999 | Significant |
| **SAN FELICE_S1_B** | **MORAIOLO_S3_D** | 0.227 | 40 | 40 | 0.001 | 999 | Significant |
| **SAN FELICE_S1_C** | **MORAIOLO_S3_D** | 0.257 | 40 | 40 | 0.001 | 999 | Significant |
| **SAN FELICE_S1_D** | **MORAIOLO_S3_D** | 0.264 | 40 | 40 | 0.001 | 999 | Significant |
| **MORAIOLO_S2_A** | **MORAIOLO_S3_D** | 0.186 | 40 | 40 | 0.001 | 999 | Significant |
| **MORAIOLO_S2_B** | **MORAIOLO_S3_D** | 0.199 | 40 | 40 | 0.001 | 999 | Significant |
| **MORAIOLO_S2_C** | **MORAIOLO_S3_D** | 0.207 | 40 | 40 | 0.001 | 999 | Significant |
| **MORAIOLO_S2_D** | **MORAIOLO_S3_D** | 0.197 | 40 | 40 | 0.001 | 999 | Significant |
| **MORAIOLO_S3_A** | **MORAIOLO_S3_D** | 0.000 | 40 | 40 | 0.431 | 999 | NS |
| **MORAIOLO_S3_B** | **MORAIOLO_S3_D** | 0.000 | 40 | 40 | 0.427 | 999 | NS |
| **MORAIOLO_S3_C** | **MORAIOLO_S3_D** | 0.000 | 40 | 40 | 0.484 | 999 | NS |
| **MORAIOLO_S1_A** | RAIO_S3_A | 0.189 | 40 | 40 | 0.001 | 999 | Significant |
| **MORAIOLO_S1_B** | RAIO_S3_A | 0.177 | 40 | 40 | 0.001 | 999 | Significant |
| **MORAIOLO_S1_C** | RAIO_S3_A | 0.193 | 40 | 40 | 0.001 | 999 | Significant |
| **MORAIOLO_S1_D** | RAIO_S3_A | 0.191 | 40 | 40 | 0.001 | 999 | Significant |
| **SAN FELICE_S1_A** | RAIO_S3_A | 0.139 | 40 | 40 | 0.001 | 999 | Significant |
| **SAN FELICE_S1_B** | RAIO_S3_A | 0.111 | 40 | 40 | 0.001 | 999 | Significant |
| **SAN FELICE_S1_C** | RAIO_S3_A | 0.142 | 40 | 40 | 0.001 | 999 | Significant |
| **SAN FELICE_S1_D** | RAIO_S3_A | 0.137 | 40 | 40 | 0.001 | 999 | Significant |
| **MORAIOLO_S2_A** | RAIO_S3_A | 0.146 | 40 | 40 | 0.001 | 999 | Significant |
| **MORAIOLO_S2_B** | RAIO_S3_A | 0.154 | 40 | 40 | 0.001 | 999 | Significant |
| **MORAIOLO_S2_C** | RAIO_S3_A | 0.173 | 40 | 40 | 0.001 | 999 | Significant |
| **MORAIOLO_S2_D** | RAIO_S3_A | 0.171 | 40 | 40 | 0.001 | 999 | Significant |
| **MORAIOLO_S3_A** | RAIO_S3_A | 0.250 | 40 | 40 | 0.001 | 999 | Significant |
| **MORAIOLO_S3_B** | RAIO_S3_A | 0.230 | 40 | 40 | 0.001 | 999 | Significant |
| **MORAIOLO_S3_C** | RAIO_S3_A | 0.267 | 40 | 40 | 0.001 | 999 | Significant |
| **MORAIOLO_S3_D** | RAIO_S3_A | 0.243 | 40 | 40 | 0.001 | 999 | Significant |
| **MORAIOLO_S1_A** | RAIO_S3_B | 0.233 | 40 | 40 | 0.001 | 999 | Significant |
| **MORAIOLO_S1_B** | RAIO_S3_B | 0.227 | 40 | 40 | 0.001 | 999 | Significant |
| **MORAIOLO_S1_C** | RAIO_S3_B | 0.235 | 40 | 40 | 0.001 | 999 | Significant |
| **MORAIOLO_S1_D** | RAIO_S3_B | 0.240 | 40 | 40 | 0.001 | 999 | Significant |
| **SAN FELICE_S1_A** | RAIO_S3_B | 0.179 | 40 | 40 | 0.001 | 999 | Significant |
| **SAN FELICE_S1_B** | RAIO_S3_B | 0.144 | 40 | 40 | 0.001 | 999 | Significant |
| **SAN FELICE_S1_C** | RAIO_S3_B | 0.180 | 40 | 40 | 0.001 | 999 | Significant |
| **SAN FELICE_S1_D** | RAIO_S3_B | 0.171 | 40 | 40 | 0.001 | 999 | Significant |
| **MORAIOLO_S2_A** | RAIO_S3_B | 0.185 | 40 | 40 | 0.001 | 999 | Significant |
| **MORAIOLO_S2_B** | RAIO_S3_B | 0.197 | 40 | 40 | 0.001 | 999 | Significant |
| **MORAIOLO_S2_C** | RAIO_S3_B | 0.217 | 40 | 40 | 0.001 | 999 | Significant |
| **MORAIOLO_S2_D** | RAIO_S3_B | 0.214 | 40 | 40 | 0.001 | 999 | Significant |
| **MORAIOLO_S3_A** | RAIO_S3_B | 0.253 | 40 | 40 | 0.001 | 999 | Significant |
| **MORAIOLO_S3_B** | RAIO_S3_B | 0.229 | 40 | 40 | 0.001 | 999 | Significant |
| **MORAIOLO_S3_C** | RAIO_S3_B | 0.273 | 40 | 40 | 0.001 | 999 | Significant |
| **MORAIOLO_S3_D** | RAIO_S3_B | 0.249 | 40 | 40 | 0.001 | 999 | Significant |
| RAIO_S3_A | RAIO_S3_B | 0.005 | 40 | 40 | 0.244 | 999 | NS |
| **MORAIOLO_S1_A** | RAIO_S3_C | 0.209 | 40 | 40 | 0.001 | 999 | Significant |
| **MORAIOLO_S1_B** | RAIO_S3_C | 0.200 | 40 | 40 | 0.001 | 999 | Significant |
| **MORAIOLO_S1_C** | RAIO_S3_C | 0.212 | 40 | 40 | 0.001 | 999 | Significant |
| **MORAIOLO_S1_D** | RAIO_S3_C | 0.220 | 40 | 40 | 0.001 | 999 | Significant |
| **SAN FELICE_S1_A** | RAIO_S3_C | 0.149 | 40 | 40 | 0.001 | 999 | Significant |
| **SAN FELICE_S1_B** | RAIO_S3_C | 0.121 | 40 | 40 | 0.001 | 999 | Significant |
| **SAN FELICE_S1_C** | RAIO_S3_C | 0.153 | 40 | 40 | 0.001 | 999 | Significant |
| **SAN FELICE_S1_D** | RAIO_S3_C | 0.147 | 40 | 40 | 0.001 | 999 | Significant |
| **MORAIOLO_S2_A** | RAIO_S3_C | 0.175 | 40 | 40 | 0.001 | 999 | Significant |
| **MORAIOLO_S2_B** | RAIO_S3_C | 0.184 | 40 | 40 | 0.001 | 999 | Significant |
| **MORAIOLO_S2_C** | RAIO_S3_C | 0.207 | 40 | 40 | 0.001 | 999 | Significant |
| **MORAIOLO_S2_D** | RAIO_S3_C | 0.207 | 40 | 40 | 0.001 | 999 | Significant |
| **MORAIOLO_S3_A** | RAIO_S3_C | 0.257 | 40 | 40 | 0.001 | 999 | Significant |
| **MORAIOLO_S3_B** | RAIO_S3_C | 0.232 | 40 | 40 | 0.001 | 999 | Significant |
| **MORAIOLO_S3_C** | RAIO_S3_C | 0.279 | 40 | 40 | 0.001 | 999 | Significant |
| **MORAIOLO_S3_D** | RAIO_S3_C | 0.251 | 40 | 40 | 0.001 | 999 | Significant |
| RAIO_S3_A | RAIO_S3_C | 0.007 | 40 | 40 | 0.193 | 999 | NS |
| RAIO_S3_B | RAIO_S3_C | 0.000 | 40 | 40 | 0.421 | 999 | NS |
| **MORAIOLO_S1_A** | RAIO_S3_D | 0.196 | 40 | 40 | 0.001 | 999 | Significant |
| **MORAIOLO_S1_B** | RAIO_S3_D | 0.183 | 40 | 40 | 0.001 | 999 | Significant |
| **MORAIOLO_S1_C** | RAIO_S3_D | 0.199 | 40 | 40 | 0.001 | 999 | Significant |
| **MORAIOLO_S1_D** | RAIO_S3_D | 0.199 | 40 | 40 | 0.001 | 999 | Significant |
| **SAN FELICE_S1_A** | RAIO_S3_D | 0.141 | 40 | 40 | 0.001 | 999 | Significant |
| **SAN FELICE_S1_B** | RAIO_S3_D | 0.116 | 40 | 40 | 0.001 | 999 | Significant |
| **SAN FELICE_S1_C** | RAIO_S3_D | 0.142 | 40 | 40 | 0.001 | 999 | Significant |
| **SAN FELICE_S1_D** | RAIO_S3_D | 0.141 | 40 | 40 | 0.001 | 999 | Significant |
| **MORAIOLO_S2_A** | RAIO_S3_D | 0.156 | 40 | 40 | 0.001 | 999 | Significant |
| **MORAIOLO_S2_B** | RAIO_S3_D | 0.164 | 40 | 40 | 0.001 | 999 | Significant |
| **MORAIOLO_S2_C** | RAIO_S3_D | 0.181 | 40 | 40 | 0.001 | 999 | Significant |
| **MORAIOLO_S2_D** | RAIO_S3_D | 0.179 | 40 | 40 | 0.001 | 999 | Significant |
| **MORAIOLO_S3_A** | RAIO_S3_D | 0.240 | 40 | 40 | 0.001 | 999 | Significant |
| **MORAIOLO_S3_B** | RAIO_S3_D | 0.218 | 40 | 40 | 0.001 | 999 | Significant |
| **MORAIOLO_S3_C** | RAIO_S3_D | 0.257 | 40 | 40 | 0.001 | 999 | Significant |
| **MORAIOLO_S3_D** | RAIO_S3_D | 0.235 | 40 | 40 | 0.001 | 999 | Significant |
| RAIO_S3_A | RAIO_S3_D | 0.000 | 40 | 40 | 0.461 | 999 | NS |
| RAIO_S3_B | RAIO_S3_D | 0.000 | 40 | 40 | 0.421 | 999 | NS |
| RAIO_S3_C | RAIO_S3_D | 0.000 | 40 | 40 | 0.395 | 999 | NS |
| **MORAIOLO_S1_A** | BORGIONA_S4_A | 0.233 | 40 | 40 | 0.001 | 999 | Significant |
| **MORAIOLO_S1_B** | BORGIONA_S4_A | 0.217 | 40 | 40 | 0.001 | 999 | Significant |
| **MORAIOLO_S1_C** | BORGIONA_S4_A | 0.228 | 40 | 40 | 0.001 | 999 | Significant |
| **MORAIOLO_S1_D** | BORGIONA_S4_A | 0.227 | 40 | 40 | 0.001 | 999 | Significant |
| **SAN FELICE_S1_A** | BORGIONA_S4_A | 0.156 | 40 | 40 | 0.001 | 999 | Significant |
| **SAN FELICE_S1_B** | BORGIONA_S4_A | 0.135 | 40 | 40 | 0.001 | 999 | Significant |
| **SAN FELICE_S1_C** | BORGIONA_S4_A | 0.159 | 40 | 40 | 0.001 | 999 | Significant |
| **SAN FELICE_S1_D** | BORGIONA_S4_A | 0.168 | 40 | 40 | 0.001 | 999 | Significant |
| **MORAIOLO_S2_A** | BORGIONA_S4_A | 0.205 | 40 | 40 | 0.001 | 999 | Significant |
| **MORAIOLO_S2_B** | BORGIONA_S4_A | 0.212 | 40 | 40 | 0.001 | 999 | Significant |
| **MORAIOLO_S2_C** | BORGIONA_S4_A | 0.233 | 40 | 40 | 0.001 | 999 | Significant |
| **MORAIOLO_S2_D** | BORGIONA_S4_A | 0.225 | 40 | 40 | 0.001 | 999 | Significant |
| **MORAIOLO_S3_A** | BORGIONA_S4_A | 0.358 | 40 | 40 | 0.001 | 999 | Significant |
| **MORAIOLO_S3_B** | BORGIONA_S4_A | 0.342 | 40 | 40 | 0.001 | 999 | Significant |
| **MORAIOLO_S3_C** | BORGIONA_S4_A | 0.363 | 40 | 40 | 0.001 | 999 | Significant |
| **MORAIOLO_S3_D** | BORGIONA_S4_A | 0.351 | 40 | 40 | 0.001 | 999 | Significant |
| RAIO_S3_A | BORGIONA_S4_A | 0.202 | 40 | 40 | 0.001 | 999 | Significant |
| RAIO_S3_B | BORGIONA_S4_A | 0.250 | 40 | 40 | 0.001 | 999 | Significant |
| RAIO_S3_C | BORGIONA_S4_A | 0.230 | 40 | 40 | 0.001 | 999 | Significant |
| RAIO_S3_D | BORGIONA_S4_A | 0.222 | 40 | 40 | 0.001 | 999 | Significant |
| **MORAIOLO_S1_A** | BORGIONA_S4_B | 0.224 | 40 | 40 | 0.001 | 999 | Significant |
| **MORAIOLO_S1_B** | BORGIONA_S4_B | 0.207 | 40 | 40 | 0.001 | 999 | Significant |
| **MORAIOLO_S1_C** | BORGIONA_S4_B | 0.222 | 40 | 40 | 0.001 | 999 | Significant |
| **MORAIOLO_S1_D** | BORGIONA_S4_B | 0.216 | 40 | 40 | 0.001 | 999 | Significant |
| **SAN FELICE_S1_A** | BORGIONA_S4_B | 0.148 | 40 | 40 | 0.001 | 999 | Significant |
| **SAN FELICE_S1_B** | BORGIONA_S4_B | 0.131 | 40 | 40 | 0.001 | 999 | Significant |
| **SAN FELICE_S1_C** | BORGIONA_S4_B | 0.149 | 40 | 40 | 0.001 | 999 | Significant |
| **SAN FELICE_S1_D** | BORGIONA_S4_B | 0.165 | 40 | 40 | 0.001 | 999 | Significant |
| **MORAIOLO_S2_A** | BORGIONA_S4_B | 0.193 | 40 | 40 | 0.001 | 999 | Significant |
| **MORAIOLO_S2_B** | BORGIONA_S4_B | 0.198 | 40 | 40 | 0.001 | 999 | Significant |
| **MORAIOLO_S2_C** | BORGIONA_S4_B | 0.215 | 40 | 40 | 0.001 | 999 | Significant |
| **MORAIOLO_S2_D** | BORGIONA_S4_B | 0.208 | 40 | 40 | 0.001 | 999 | Significant |
| **MORAIOLO_S3_A** | BORGIONA_S4_B | 0.347 | 40 | 40 | 0.001 | 999 | Significant |
| **MORAIOLO_S3_B** | BORGIONA_S4_B | 0.332 | 40 | 40 | 0.001 | 999 | Significant |
| **MORAIOLO_S3_C** | BORGIONA_S4_B | 0.351 | 40 | 40 | 0.001 | 999 | Significant |
| **MORAIOLO_S3_D** | BORGIONA_S4_B | 0.340 | 40 | 40 | 0.001 | 999 | Significant |
| RAIO_S3_A | BORGIONA_S4_B | 0.188 | 40 | 40 | 0.001 | 999 | Significant |
| RAIO_S3_B | BORGIONA_S4_B | 0.237 | 40 | 40 | 0.001 | 999 | Significant |
| RAIO_S3_C | BORGIONA_S4_B | 0.221 | 40 | 40 | 0.001 | 999 | Significant |
| RAIO_S3_D | BORGIONA_S4_B | 0.202 | 40 | 40 | 0.001 | 999 | Significant |
| BORGIONA_S4_A | BORGIONA_S4_B | 0.000 | 40 | 40 | 0.412 | 999 | NS |
| **MORAIOLO_S1_A** | BORGIONA_S4_C | 0.211 | 40 | 40 | 0.001 | 999 | Significant |
| **MORAIOLO_S1_B** | BORGIONA_S4_C | 0.196 | 40 | 40 | 0.001 | 999 | Significant |
| **MORAIOLO_S1_C** | BORGIONA_S4_C | 0.204 | 40 | 40 | 0.001 | 999 | Significant |
| **MORAIOLO_S1_D** | BORGIONA_S4_C | 0.207 | 40 | 40 | 0.001 | 999 | Significant |
| **SAN FELICE_S1_A** | BORGIONA_S4_C | 0.134 | 40 | 40 | 0.001 | 999 | Significant |
| **SAN FELICE_S1_B** | BORGIONA_S4_C | 0.105 | 40 | 40 | 0.001 | 999 | Significant |
| **SAN FELICE_S1_C** | BORGIONA_S4_C | 0.135 | 40 | 40 | 0.001 | 999 | Significant |
| **SAN FELICE_S1_D** | BORGIONA_S4_C | 0.137 | 40 | 40 | 0.001 | 999 | Significant |
| **MORAIOLO_S2_A** | BORGIONA_S4_C | 0.170 | 40 | 40 | 0.001 | 999 | Significant |
| **MORAIOLO_S2_B** | BORGIONA_S4_C | 0.178 | 40 | 40 | 0.001 | 999 | Significant |
| **MORAIOLO_S2_C** | BORGIONA_S4_C | 0.197 | 40 | 40 | 0.001 | 999 | Significant |
| **MORAIOLO_S2_D** | BORGIONA_S4_C | 0.188 | 40 | 40 | 0.001 | 999 | Significant |
| **MORAIOLO_S3_A** | BORGIONA_S4_C | 0.304 | 40 | 40 | 0.001 | 999 | Significant |
| **MORAIOLO_S3_B** | BORGIONA_S4_C | 0.286 | 40 | 40 | 0.001 | 999 | Significant |
| **MORAIOLO_S3_C** | BORGIONA_S4_C | 0.310 | 40 | 40 | 0.001 | 999 | Significant |
| **MORAIOLO_S3_D** | BORGIONA_S4_C | 0.297 | 40 | 40 | 0.001 | 999 | Significant |
| RAIO_S3_A | BORGIONA_S4_C | 0.140 | 40 | 40 | 0.001 | 999 | Significant |
| RAIO_S3_B | BORGIONA_S4_C | 0.170 | 40 | 40 | 0.001 | 999 | Significant |
| RAIO_S3_C | BORGIONA_S4_C | 0.159 | 40 | 40 | 0.001 | 999 | Significant |
| RAIO_S3_D | BORGIONA_S4_C | 0.150 | 40 | 40 | 0.001 | 999 | Significant |
| BORGIONA_S4_A | BORGIONA_S4_C | 0.005 | 40 | 40 | 0.254 | 999 | NS |
| BORGIONA_S4_B | BORGIONA_S4_C | 0.000 | 40 | 40 | 0.442 | 999 | NS |
| **MORAIOLO_S1_A** | BORGIONA_S4_D | 0.231 | 40 | 40 | 0.001 | 999 | Significant |
| **MORAIOLO_S1_B** | BORGIONA_S4_D | 0.212 | 40 | 40 | 0.001 | 999 | Significant |
| **MORAIOLO_S1_C** | BORGIONA_S4_D | 0.224 | 40 | 40 | 0.001 | 999 | Significant |
| **MORAIOLO_S1_D** | BORGIONA_S4_D | 0.220 | 40 | 40 | 0.001 | 999 | Significant |
| **SAN FELICE_S1_A** | BORGIONA_S4_D | 0.158 | 40 | 40 | 0.001 | 999 | Significant |
| **SAN FELICE_S1_B** | BORGIONA_S4_D | 0.136 | 40 | 40 | 0.001 | 999 | Significant |
| **SAN FELICE_S1_C** | BORGIONA_S4_D | 0.156 | 40 | 40 | 0.001 | 999 | Significant |
| **SAN FELICE_S1_D** | BORGIONA_S4_D | 0.168 | 40 | 40 | 0.001 | 999 | Significant |
| **MORAIOLO_S2_A** | BORGIONA_S4_D | 0.192 | 40 | 40 | 0.001 | 999 | Significant |
| **MORAIOLO_S2_B** | BORGIONA_S4_D | 0.199 | 40 | 40 | 0.001 | 999 | Significant |
| **MORAIOLO_S2_C** | BORGIONA_S4_D | 0.216 | 40 | 40 | 0.001 | 999 | Significant |
| **MORAIOLO_S2_D** | BORGIONA_S4_D | 0.208 | 40 | 40 | 0.001 | 999 | Significant |
| **MORAIOLO_S3_A** | BORGIONA_S4_D | 0.342 | 40 | 40 | 0.001 | 999 | Significant |
| **MORAIOLO_S3_B** | BORGIONA_S4_D | 0.329 | 40 | 40 | 0.001 | 999 | Significant |
| **MORAIOLO_S3_C** | BORGIONA_S4_D | 0.350 | 40 | 40 | 0.001 | 999 | Significant |
| **MORAIOLO_S3_D** | BORGIONA_S4_D | 0.337 | 40 | 40 | 0.001 | 999 | Significant |
| RAIO_S3_A | BORGIONA_S4_D | 0.175 | 40 | 40 | 0.001 | 999 | Significant |
| RAIO_S3_B | BORGIONA_S4_D | 0.219 | 40 | 40 | 0.001 | 999 | Significant |
| RAIO_S3_C | BORGIONA_S4_D | 0.205 | 40 | 40 | 0.001 | 999 | Significant |
| RAIO_S3_D | BORGIONA_S4_D | 0.191 | 40 | 40 | 0.001 | 999 | Significant |
| BORGIONA_S4_A | BORGIONA_S4_D | 0.000 | 40 | 40 | 0.419 | 999 | NS |
| BORGIONA_S4_B | BORGIONA_S4_D | 0.000 | 40 | 40 | 0.439 | 999 | NS |
| BORGIONA_S4_C | BORGIONA_S4_D | 0.000 | 40 | 40 | 0.443 | 999 | NS |
| **MORAIOLO_S1_A** | **GENTILE GRANDE_S4_A** | 0.220 | 40 | 40 | 0.001 | 999 | Significant |
| **MORAIOLO_S1_B** | **GENTILE GRANDE_S4_A** | 0.209 | 40 | 40 | 0.001 | 999 | Significant |
| **MORAIOLO_S1_C** | **GENTILE GRANDE_S4_A** | 0.215 | 40 | 40 | 0.001 | 999 | Significant |
| **MORAIOLO_S1_D** | **GENTILE GRANDE_S4_A** | 0.217 | 40 | 40 | 0.001 | 999 | Significant |
| **SAN FELICE_S1_A** | **GENTILE GRANDE_S4_A** | 0.160 | 40 | 40 | 0.001 | 999 | Significant |
| **SAN FELICE_S1_B** | **GENTILE GRANDE_S4_A** | 0.113 | 40 | 40 | 0.001 | 999 | Significant |
| **SAN FELICE_S1_C** | **GENTILE GRANDE_S4_A** | 0.161 | 40 | 40 | 0.001 | 999 | Significant |
| **SAN FELICE_S1_D** | **GENTILE GRANDE_S4_A** | 0.168 | 40 | 40 | 0.001 | 999 | Significant |
| **MORAIOLO_S2_A** | **GENTILE GRANDE_S4_A** | 0.111 | 40 | 40 | 0.001 | 999 | Significant |
| **MORAIOLO_S2_B** | **GENTILE GRANDE_S4_A** | 0.123 | 40 | 40 | 0.001 | 999 | Significant |
| **MORAIOLO_S2_C** | **GENTILE GRANDE_S4_A** | 0.132 | 40 | 40 | 0.001 | 999 | Significant |
| **MORAIOLO_S2_D** | **GENTILE GRANDE_S4_A** | 0.124 | 40 | 40 | 0.001 | 999 | Significant |
| **MORAIOLO_S3_A** | **GENTILE GRANDE_S4_A** | 0.240 | 40 | 40 | 0.001 | 999 | Significant |
| **MORAIOLO_S3_B** | **GENTILE GRANDE_S4_A** | 0.219 | 40 | 40 | 0.001 | 999 | Significant |
| **MORAIOLO_S3_C** | **GENTILE GRANDE_S4_A** | 0.240 | 40 | 40 | 0.001 | 999 | Significant |
| **MORAIOLO_S3_D** | **GENTILE GRANDE_S4_A** | 0.235 | 40 | 40 | 0.001 | 999 | Significant |
| RAIO_S3_A | **GENTILE GRANDE_S4_A** | 0.162 | 40 | 40 | 0.001 | 999 | Significant |
| RAIO_S3_B | **GENTILE GRANDE_S4_A** | 0.195 | 40 | 40 | 0.001 | 999 | Significant |
| RAIO_S3_C | **GENTILE GRANDE_S4_A** | 0.204 | 40 | 40 | 0.001 | 999 | Significant |
| RAIO_S3_D | **GENTILE GRANDE_S4_A** | 0.170 | 40 | 40 | 0.001 | 999 | Significant |
| BORGIONA_S4_A | **GENTILE GRANDE_S4_A** | 0.232 | 40 | 40 | 0.001 | 999 | Significant |
| BORGIONA_S4_B | **GENTILE GRANDE_S4_A** | 0.215 | 40 | 40 | 0.001 | 999 | Significant |
| BORGIONA_S4_C | **GENTILE GRANDE_S4_A** | 0.186 | 40 | 40 | 0.001 | 999 | Significant |
| BORGIONA_S4_D | **GENTILE GRANDE_S4_A** | 0.225 | 40 | 40 | 0.001 | 999 | Significant |
| **MORAIOLO_S1_A** | **GENTILE GRANDE_S4_B** | 0.184 | 40 | 40 | 0.001 | 999 | Significant |
| **MORAIOLO_S1_B** | **GENTILE GRANDE_S4_B** | 0.178 | 40 | 40 | 0.001 | 999 | Significant |
| **MORAIOLO_S1_C** | **GENTILE GRANDE_S4_B** | 0.178 | 40 | 40 | 0.001 | 999 | Significant |
| **MORAIOLO_S1_D** | **GENTILE GRANDE_S4_B** | 0.183 | 40 | 40 | 0.001 | 999 | Significant |
| **SAN FELICE_S1_A** | **GENTILE GRANDE_S4_B** | 0.131 | 40 | 40 | 0.001 | 999 | Significant |
| **SAN FELICE_S1_B** | **GENTILE GRANDE_S4_B** | 0.094 | 40 | 40 | 0.001 | 999 | Significant |
| **SAN FELICE_S1_C** | **GENTILE GRANDE_S4_B** | 0.130 | 40 | 40 | 0.001 | 999 | Significant |
| **SAN FELICE_S1_D** | **GENTILE GRANDE_S4_B** | 0.136 | 40 | 40 | 0.001 | 999 | Significant |
| **MORAIOLO_S2_A** | **GENTILE GRANDE_S4_B** | 0.083 | 40 | 40 | 0.001 | 999 | Significant |
| **MORAIOLO_S2_B** | **GENTILE GRANDE_S4_B** | 0.094 | 40 | 40 | 0.001 | 999 | Significant |
| **MORAIOLO_S2_C** | **GENTILE GRANDE_S4_B** | 0.103 | 40 | 40 | 0.001 | 999 | Significant |
| **MORAIOLO_S2_D** | **GENTILE GRANDE_S4_B** | 0.100 | 40 | 40 | 0.001 | 999 | Significant |
| **MORAIOLO_S3_A** | **GENTILE GRANDE_S4_B** | 0.209 | 40 | 40 | 0.001 | 999 | Significant |
| **MORAIOLO_S3_B** | **GENTILE GRANDE_S4_B** | 0.195 | 40 | 40 | 0.001 | 999 | Significant |
| **MORAIOLO_S3_C** | **GENTILE GRANDE_S4_B** | 0.215 | 40 | 40 | 0.001 | 999 | Significant |
| **MORAIOLO_S3_D** | **GENTILE GRANDE_S4_B** | 0.207 | 40 | 40 | 0.001 | 999 | Significant |
| RAIO_S3_A | **GENTILE GRANDE_S4_B** | 0.126 | 40 | 40 | 0.001 | 999 | Significant |
| RAIO_S3_B | **GENTILE GRANDE_S4_B** | 0.156 | 40 | 40 | 0.001 | 999 | Significant |
| RAIO_S3_C | **GENTILE GRANDE_S4_B** | 0.160 | 40 | 40 | 0.001 | 999 | Significant |
| RAIO_S3_D | **GENTILE GRANDE_S4_B** | 0.128 | 40 | 40 | 0.001 | 999 | Significant |
| BORGIONA_S4_A | **GENTILE GRANDE_S4_B** | 0.196 | 40 | 40 | 0.001 | 999 | Significant |
| BORGIONA_S4_B | **GENTILE GRANDE_S4_B** | 0.178 | 40 | 40 | 0.001 | 999 | Significant |
| BORGIONA_S4_C | **GENTILE GRANDE_S4_B** | 0.147 | 40 | 40 | 0.001 | 999 | Significant |
| BORGIONA_S4_D | **GENTILE GRANDE_S4_B** | 0.182 | 40 | 40 | 0.001 | 999 | Significant |
| **GENTILE GRANDE_S4_A** | **GENTILE GRANDE_S4_B** | 0.024 | 40 | 40 | 0.013 | 999 | Significant |
| **MORAIOLO_S1_A** | **GENTILE GRANDE_S4_C** | 0.216 | 40 | 40 | 0.001 | 999 | Significant |
| **MORAIOLO_S1_B** | **GENTILE GRANDE_S4_C** | 0.206 | 40 | 40 | 0.001 | 999 | Significant |
| **MORAIOLO_S1_C** | **GENTILE GRANDE_S4_C** | 0.212 | 40 | 40 | 0.001 | 999 | Significant |
| **MORAIOLO_S1_D** | **GENTILE GRANDE_S4_C** | 0.213 | 40 | 40 | 0.001 | 999 | Significant |
| **SAN FELICE_S1_A** | **GENTILE GRANDE_S4_C** | 0.156 | 40 | 40 | 0.001 | 999 | Significant |
| **SAN FELICE_S1_B** | **GENTILE GRANDE_S4_C** | 0.110 | 40 | 40 | 0.001 | 999 | Significant |
| **SAN FELICE_S1_C** | **GENTILE GRANDE_S4_C** | 0.158 | 40 | 40 | 0.001 | 999 | Significant |
| **SAN FELICE_S1_D** | **GENTILE GRANDE_S4_C** | 0.163 | 40 | 40 | 0.001 | 999 | Significant |
| **MORAIOLO_S2_A** | **GENTILE GRANDE_S4_C** | 0.113 | 40 | 40 | 0.001 | 999 | Significant |
| **MORAIOLO_S2_B** | **GENTILE GRANDE_S4_C** | 0.125 | 40 | 40 | 0.001 | 999 | Significant |
| **MORAIOLO_S2_C** | **GENTILE GRANDE_S4_C** | 0.135 | 40 | 40 | 0.001 | 999 | Significant |
| **MORAIOLO_S2_D** | **GENTILE GRANDE_S4_C** | 0.127 | 40 | 40 | 0.001 | 999 | Significant |
| **MORAIOLO_S3_A** | **GENTILE GRANDE_S4_C** | 0.222 | 40 | 40 | 0.001 | 999 | Significant |
| **MORAIOLO_S3_B** | **GENTILE GRANDE_S4_C** | 0.204 | 40 | 40 | 0.001 | 999 | Significant |
| **MORAIOLO_S3_C** | **GENTILE GRANDE_S4_C** | 0.224 | 40 | 40 | 0.001 | 999 | Significant |
| **MORAIOLO_S3_D** | **GENTILE GRANDE_S4_C** | 0.221 | 40 | 40 | 0.001 | 999 | Significant |
| RAIO_S3_A | **GENTILE GRANDE_S4_C** | 0.152 | 40 | 40 | 0.001 | 999 | Significant |
| RAIO_S3_B | **GENTILE GRANDE_S4_C** | 0.180 | 40 | 40 | 0.001 | 999 | Significant |
| RAIO_S3_C | **GENTILE GRANDE_S4_C** | 0.189 | 40 | 40 | 0.001 | 999 | Significant |
| RAIO_S3_D | **GENTILE GRANDE_S4_C** | 0.156 | 40 | 40 | 0.001 | 999 | Significant |
| BORGIONA_S4_A | **GENTILE GRANDE_S4_C** | 0.222 | 40 | 40 | 0.001 | 999 | Significant |
| BORGIONA_S4_B | **GENTILE GRANDE_S4_C** | 0.209 | 40 | 40 | 0.001 | 999 | Significant |
| BORGIONA_S4_C | **GENTILE GRANDE_S4_C** | 0.179 | 40 | 40 | 0.001 | 999 | Significant |
| BORGIONA_S4_D | **GENTILE GRANDE_S4_C** | 0.217 | 40 | 40 | 0.001 | 999 | Significant |
| **GENTILE GRANDE_S4_A** | **GENTILE GRANDE_S4_C** | 0.000 | 40 | 40 | 0.388 | 999 | NS |
| **GENTILE GRANDE_S4_B** | **GENTILE GRANDE_S4_C** | 0.007 | 40 | 40 | 0.198 | 999 | NS |
| **MORAIOLO_S1_A** | **GENTILE GRANDE_S4_D** | 0.192 | 40 | 40 | 0.001 | 999 | Significant |
| **MORAIOLO_S1_B** | **GENTILE GRANDE_S4_D** | 0.184 | 40 | 40 | 0.001 | 999 | Significant |
| **MORAIOLO_S1_C** | **GENTILE GRANDE_S4_D** | 0.185 | 40 | 40 | 0.001 | 999 | Significant |
| **MORAIOLO_S1_D** | **GENTILE GRANDE_S4_D** | 0.185 | 40 | 40 | 0.001 | 999 | Significant |
| **SAN FELICE_S1_A** | **GENTILE GRANDE_S4_D** | 0.144 | 40 | 40 | 0.001 | 999 | Significant |
| **SAN FELICE_S1_B** | **GENTILE GRANDE_S4_D** | 0.096 | 40 | 40 | 0.001 | 999 | Significant |
| **SAN FELICE_S1_C** | **GENTILE GRANDE_S4_D** | 0.141 | 40 | 40 | 0.001 | 999 | Significant |
| **SAN FELICE_S1_D** | **GENTILE GRANDE_S4_D** | 0.146 | 40 | 40 | 0.001 | 999 | Significant |
| **MORAIOLO_S2_A** | **GENTILE GRANDE_S4_D** | 0.096 | 40 | 40 | 0.001 | 999 | Significant |
| **MORAIOLO_S2_B** | **GENTILE GRANDE_S4_D** | 0.112 | 40 | 40 | 0.001 | 999 | Significant |
| **MORAIOLO_S2_C** | **GENTILE GRANDE_S4_D** | 0.121 | 40 | 40 | 0.001 | 999 | Significant |
| **MORAIOLO_S2_D** | **GENTILE GRANDE_S4_D** | 0.115 | 40 | 40 | 0.001 | 999 | Significant |
| **MORAIOLO_S3_A** | **GENTILE GRANDE_S4_D** | 0.219 | 40 | 40 | 0.001 | 999 | Significant |
| **MORAIOLO_S3_B** | **GENTILE GRANDE_S4_D** | 0.201 | 40 | 40 | 0.001 | 999 | Significant |
| **MORAIOLO_S3_C** | **GENTILE GRANDE_S4_D** | 0.223 | 40 | 40 | 0.001 | 999 | Significant |
| **MORAIOLO_S3_D** | **GENTILE GRANDE_S4_D** | 0.218 | 40 | 40 | 0.001 | 999 | Significant |
| RAIO_S3_A | **GENTILE GRANDE_S4_D** | 0.139 | 40 | 40 | 0.001 | 999 | Significant |
| RAIO_S3_B | **GENTILE GRANDE_S4_D** | 0.170 | 40 | 40 | 0.001 | 999 | Significant |
| RAIO_S3_C | **GENTILE GRANDE_S4_D** | 0.182 | 40 | 40 | 0.001 | 999 | Significant |
| RAIO_S3_D | **GENTILE GRANDE_S4_D** | 0.148 | 40 | 40 | 0.001 | 999 | Significant |
| BORGIONA_S4_A | **GENTILE GRANDE_S4_D** | 0.213 | 40 | 40 | 0.001 | 999 | Significant |
| BORGIONA_S4_B | **GENTILE GRANDE_S4_D** | 0.201 | 40 | 40 | 0.001 | 999 | Significant |
| BORGIONA_S4_C | **GENTILE GRANDE_S4_D** | 0.171 | 40 | 40 | 0.001 | 999 | Significant |
| BORGIONA_S4_D | **GENTILE GRANDE_S4_D** | 0.205 | 40 | 40 | 0.001 | 999 | Significant |
| **GENTILE GRANDE_S4_A** | **GENTILE GRANDE_S4_D** | 0.000 | 40 | 40 | 0.422 | 999 | NS |
| **GENTILE GRANDE_S4_B** | **GENTILE GRANDE_S4_D** | 0.010 | 40 | 40 | 0.096 | 999 | NS |
| **GENTILE GRANDE_S4_C** | **GENTILE GRANDE_S4_D** | 0.000 | 40 | 40 | 0.433 | 999 | NS |
| **MORAIOLO_S1_A** | DOLCE AGOGIA_S5_A | 0.160 | 40 | 40 | 0.001 | 999 | Significant |
| **MORAIOLO_S1_B** | DOLCE AGOGIA_S5_A | 0.150 | 40 | 40 | 0.001 | 999 | Significant |
| **MORAIOLO_S1_C** | DOLCE AGOGIA_S5_A | 0.161 | 40 | 40 | 0.001 | 999 | Significant |
| **MORAIOLO_S1_D** | DOLCE AGOGIA_S5_A | 0.162 | 40 | 40 | 0.001 | 999 | Significant |
| **SAN FELICE_S1_A** | DOLCE AGOGIA_S5_A | 0.106 | 40 | 40 | 0.001 | 999 | Significant |
| **SAN FELICE_S1_B** | DOLCE AGOGIA_S5_A | 0.084 | 40 | 40 | 0.001 | 999 | Significant |
| **SAN FELICE_S1_C** | DOLCE AGOGIA_S5_A | 0.110 | 40 | 40 | 0.001 | 999 | Significant |
| **SAN FELICE_S1_D** | DOLCE AGOGIA_S5_A | 0.119 | 40 | 40 | 0.001 | 999 | Significant |
| **MORAIOLO_S2_A** | DOLCE AGOGIA_S5_A | 0.084 | 40 | 40 | 0.001 | 999 | Significant |
| **MORAIOLO_S2_B** | DOLCE AGOGIA_S5_A | 0.097 | 40 | 40 | 0.001 | 999 | Significant |
| **MORAIOLO_S2_C** | DOLCE AGOGIA_S5_A | 0.098 | 40 | 40 | 0.001 | 999 | Significant |
| **MORAIOLO_S2_D** | DOLCE AGOGIA_S5_A | 0.100 | 40 | 40 | 0.001 | 999 | Significant |
| **MORAIOLO_S3_A** | DOLCE AGOGIA_S5_A | 0.197 | 40 | 40 | 0.001 | 999 | Significant |
| **MORAIOLO_S3_B** | DOLCE AGOGIA_S5_A | 0.176 | 40 | 40 | 0.001 | 999 | Significant |
| **MORAIOLO_S3_C** | DOLCE AGOGIA_S5_A | 0.201 | 40 | 40 | 0.001 | 999 | Significant |
| **MORAIOLO_S3_D** | DOLCE AGOGIA_S5_A | 0.190 | 40 | 40 | 0.001 | 999 | Significant |
| RAIO_S3_A | DOLCE AGOGIA_S5_A | 0.100 | 40 | 40 | 0.001 | 999 | Significant |
| RAIO_S3_B | DOLCE AGOGIA_S5_A | 0.124 | 40 | 40 | 0.001 | 999 | Significant |
| RAIO_S3_C | DOLCE AGOGIA_S5_A | 0.126 | 40 | 40 | 0.001 | 999 | Significant |
| RAIO_S3_D | DOLCE AGOGIA_S5_A | 0.096 | 40 | 40 | 0.001 | 999 | Significant |
| BORGIONA_S4_A | DOLCE AGOGIA_S5_A | 0.190 | 40 | 40 | 0.001 | 999 | Significant |
| BORGIONA_S4_B | DOLCE AGOGIA_S5_A | 0.169 | 40 | 40 | 0.001 | 999 | Significant |
| BORGIONA_S4_C | DOLCE AGOGIA_S5_A | 0.135 | 40 | 40 | 0.001 | 999 | Significant |
| BORGIONA_S4_D | DOLCE AGOGIA_S5_A | 0.170 | 40 | 40 | 0.001 | 999 | Significant |
| **GENTILE GRANDE_S4_A** | DOLCE AGOGIA_S5_A | 0.096 | 40 | 40 | 0.001 | 999 | Significant |
| **GENTILE GRANDE_S4_B** | DOLCE AGOGIA_S5_A | 0.073 | 40 | 40 | 0.001 | 999 | Significant |
| **GENTILE GRANDE_S4_C** | DOLCE AGOGIA_S5_A | 0.089 | 40 | 40 | 0.001 | 999 | Significant |
| **GENTILE GRANDE_S4_D** | DOLCE AGOGIA_S5_A | 0.083 | 40 | 40 | 0.001 | 999 | Significant |
| **MORAIOLO_S1_A** | DOLCE AGOGIA_S5_B | 0.176 | 40 | 40 | 0.001 | 999 | Significant |
| **MORAIOLO_S1_B** | DOLCE AGOGIA_S5_B | 0.170 | 40 | 40 | 0.001 | 999 | Significant |
| **MORAIOLO_S1_C** | DOLCE AGOGIA_S5_B | 0.178 | 40 | 40 | 0.001 | 999 | Significant |
| **MORAIOLO_S1_D** | DOLCE AGOGIA_S5_B | 0.179 | 40 | 40 | 0.001 | 999 | Significant |
| **SAN FELICE_S1_A** | DOLCE AGOGIA_S5_B | 0.117 | 40 | 40 | 0.001 | 999 | Significant |
| **SAN FELICE_S1_B** | DOLCE AGOGIA_S5_B | 0.087 | 40 | 40 | 0.001 | 999 | Significant |
| **SAN FELICE_S1_C** | DOLCE AGOGIA_S5_B | 0.119 | 40 | 40 | 0.001 | 999 | Significant |
| **SAN FELICE_S1_D** | DOLCE AGOGIA_S5_B | 0.128 | 40 | 40 | 0.001 | 999 | Significant |
| **MORAIOLO_S2_A** | DOLCE AGOGIA_S5_B | 0.089 | 40 | 40 | 0.001 | 999 | Significant |
| **MORAIOLO_S2_B** | DOLCE AGOGIA_S5_B | 0.105 | 40 | 40 | 0.001 | 999 | Significant |
| **MORAIOLO_S2_C** | DOLCE AGOGIA_S5_B | 0.107 | 40 | 40 | 0.001 | 999 | Significant |
| **MORAIOLO_S2_D** | DOLCE AGOGIA_S5_B | 0.108 | 40 | 40 | 0.001 | 999 | Significant |
| **MORAIOLO_S3_A** | DOLCE AGOGIA_S5_B | 0.197 | 40 | 40 | 0.001 | 999 | Significant |
| **MORAIOLO_S3_B** | DOLCE AGOGIA_S5_B | 0.177 | 40 | 40 | 0.001 | 999 | Significant |
| **MORAIOLO_S3_C** | DOLCE AGOGIA_S5_B | 0.197 | 40 | 40 | 0.001 | 999 | Significant |
| **MORAIOLO_S3_D** | DOLCE AGOGIA_S5_B | 0.187 | 40 | 40 | 0.001 | 999 | Significant |
| RAIO_S3_A | DOLCE AGOGIA_S5_B | 0.103 | 40 | 40 | 0.001 | 999 | Significant |
| RAIO_S3_B | DOLCE AGOGIA_S5_B | 0.128 | 40 | 40 | 0.001 | 999 | Significant |
| RAIO_S3_C | DOLCE AGOGIA_S5_B | 0.135 | 40 | 40 | 0.001 | 999 | Significant |
| RAIO_S3_D | DOLCE AGOGIA_S5_B | 0.102 | 40 | 40 | 0.001 | 999 | Significant |
| BORGIONA_S4_A | DOLCE AGOGIA_S5_B | 0.193 | 40 | 40 | 0.001 | 999 | Significant |
| BORGIONA_S4_B | DOLCE AGOGIA_S5_B | 0.174 | 40 | 40 | 0.001 | 999 | Significant |
| BORGIONA_S4_C | DOLCE AGOGIA_S5_B | 0.140 | 40 | 40 | 0.001 | 999 | Significant |
| BORGIONA_S4_D | DOLCE AGOGIA_S5_B | 0.179 | 40 | 40 | 0.001 | 999 | Significant |
| **GENTILE GRANDE_S4_A** | DOLCE AGOGIA_S5_B | 0.082 | 40 | 40 | 0.001 | 999 | Significant |
| **GENTILE GRANDE_S4_B** | DOLCE AGOGIA_S5_B | 0.065 | 40 | 40 | 0.001 | 999 | Significant |
| **GENTILE GRANDE_S4_C** | DOLCE AGOGIA_S5_B | 0.075 | 40 | 40 | 0.001 | 999 | Significant |
| **GENTILE GRANDE_S4_D** | DOLCE AGOGIA_S5_B | 0.070 | 40 | 40 | 0.001 | 999 | Significant |
| DOLCE AGOGIA_S5_A | DOLCE AGOGIA_S5_B | 0.000 | 40 | 40 | 0.460 | 999 | NS |
| **MORAIOLO_S1_A** | DOLCE AGOGIA_S5_C | 0.158 | 40 | 40 | 0.001 | 999 | Significant |
| **MORAIOLO_S1_B** | DOLCE AGOGIA_S5_C | 0.151 | 40 | 40 | 0.001 | 999 | Significant |
| **MORAIOLO_S1_C** | DOLCE AGOGIA_S5_C | 0.159 | 40 | 40 | 0.001 | 999 | Significant |
| **MORAIOLO_S1_D** | DOLCE AGOGIA_S5_C | 0.158 | 40 | 40 | 0.001 | 999 | Significant |
| **SAN FELICE_S1_A** | DOLCE AGOGIA_S5_C | 0.110 | 40 | 40 | 0.001 | 999 | Significant |
| **SAN FELICE_S1_B** | DOLCE AGOGIA_S5_C | 0.076 | 40 | 40 | 0.001 | 999 | Significant |
| **SAN FELICE_S1_C** | DOLCE AGOGIA_S5_C | 0.110 | 40 | 40 | 0.001 | 999 | Significant |
| **SAN FELICE_S1_D** | DOLCE AGOGIA_S5_C | 0.113 | 40 | 40 | 0.001 | 999 | Significant |
| **MORAIOLO_S2_A** | DOLCE AGOGIA_S5_C | 0.089 | 40 | 40 | 0.001 | 999 | Significant |
| **MORAIOLO_S2_B** | DOLCE AGOGIA_S5_C | 0.102 | 40 | 40 | 0.001 | 999 | Significant |
| **MORAIOLO_S2_C** | DOLCE AGOGIA_S5_C | 0.110 | 40 | 40 | 0.001 | 999 | Significant |
| **MORAIOLO_S2_D** | DOLCE AGOGIA_S5_C | 0.112 | 40 | 40 | 0.001 | 999 | Significant |
| **MORAIOLO_S3_A** | DOLCE AGOGIA_S5_C | 0.210 | 40 | 40 | 0.001 | 999 | Significant |
| **MORAIOLO_S3_B** | DOLCE AGOGIA_S5_C | 0.188 | 40 | 40 | 0.001 | 999 | Significant |
| **MORAIOLO_S3_C** | DOLCE AGOGIA_S5_C | 0.212 | 40 | 40 | 0.001 | 999 | Significant |
| **MORAIOLO_S3_D** | DOLCE AGOGIA_S5_C | 0.201 | 40 | 40 | 0.001 | 999 | Significant |
| RAIO_S3_A | DOLCE AGOGIA_S5_C | 0.095 | 40 | 40 | 0.001 | 999 | Significant |
| RAIO_S3_B | DOLCE AGOGIA_S5_C | 0.123 | 40 | 40 | 0.001 | 999 | Significant |
| RAIO_S3_C | DOLCE AGOGIA_S5_C | 0.129 | 40 | 40 | 0.001 | 999 | Significant |
| RAIO_S3_D | DOLCE AGOGIA_S5_C | 0.098 | 40 | 40 | 0.001 | 999 | Significant |
| BORGIONA_S4_A | DOLCE AGOGIA_S5_C | 0.190 | 40 | 40 | 0.001 | 999 | Significant |
| BORGIONA_S4_B | DOLCE AGOGIA_S5_C | 0.175 | 40 | 40 | 0.001 | 999 | Significant |
| BORGIONA_S4_C | DOLCE AGOGIA_S5_C | 0.139 | 40 | 40 | 0.001 | 999 | Significant |
| BORGIONA_S4_D | DOLCE AGOGIA_S5_C | 0.180 | 40 | 40 | 0.001 | 999 | Significant |
| **GENTILE GRANDE_S4_A** | DOLCE AGOGIA_S5_C | 0.087 | 40 | 40 | 0.001 | 999 | Significant |
| **GENTILE GRANDE_S4_B** | DOLCE AGOGIA_S5_C | 0.071 | 40 | 40 | 0.001 | 999 | Significant |
| **GENTILE GRANDE_S4_C** | DOLCE AGOGIA_S5_C | 0.081 | 40 | 40 | 0.001 | 999 | Significant |
| **GENTILE GRANDE_S4_D** | DOLCE AGOGIA_S5_C | 0.071 | 40 | 40 | 0.001 | 999 | Significant |
| DOLCE AGOGIA_S5_A | DOLCE AGOGIA_S5_C | 0.000 | 40 | 40 | 0.424 | 999 | NS |
| DOLCE AGOGIA_S5_B | DOLCE AGOGIA_S5_C | 0.000 | 40 | 40 | 0.421 | 999 | NS |
| **MORAIOLO_S1_A** | DOLCE AGOGIA_S5_D | 0.139 | 40 | 40 | 0.001 | 999 | Significant |
| **MORAIOLO_S1_B** | DOLCE AGOGIA_S5_D | 0.129 | 40 | 40 | 0.001 | 999 | Significant |
| **MORAIOLO_S1_C** | DOLCE AGOGIA_S5_D | 0.141 | 40 | 40 | 0.001 | 999 | Significant |
| **MORAIOLO_S1_D** | DOLCE AGOGIA_S5_D | 0.143 | 40 | 40 | 0.001 | 999 | Significant |
| **SAN FELICE_S1_A** | DOLCE AGOGIA_S5_D | 0.084 | 40 | 40 | 0.001 | 999 | Significant |
| **SAN FELICE_S1_B** | DOLCE AGOGIA_S5_D | 0.064 | 40 | 40 | 0.001 | 999 | Significant |
| **SAN FELICE_S1_C** | DOLCE AGOGIA_S5_D | 0.087 | 40 | 40 | 0.001 | 999 | Significant |
| **SAN FELICE_S1_D** | DOLCE AGOGIA_S5_D | 0.098 | 40 | 40 | 0.001 | 999 | Significant |
| **MORAIOLO_S2_A** | DOLCE AGOGIA_S5_D | 0.085 | 40 | 40 | 0.001 | 999 | Significant |
| **MORAIOLO_S2_B** | DOLCE AGOGIA_S5_D | 0.098 | 40 | 40 | 0.001 | 999 | Significant |
| **MORAIOLO_S2_C** | DOLCE AGOGIA_S5_D | 0.104 | 40 | 40 | 0.001 | 999 | Significant |
| **MORAIOLO_S2_D** | DOLCE AGOGIA_S5_D | 0.105 | 40 | 40 | 0.001 | 999 | Significant |
| **MORAIOLO_S3_A** | DOLCE AGOGIA_S5_D | 0.197 | 40 | 40 | 0.001 | 999 | Significant |
| **MORAIOLO_S3_B** | DOLCE AGOGIA_S5_D | 0.176 | 40 | 40 | 0.001 | 999 | Significant |
| **MORAIOLO_S3_C** | DOLCE AGOGIA_S5_D | 0.201 | 40 | 40 | 0.001 | 999 | Significant |
| **MORAIOLO_S3_D** | DOLCE AGOGIA_S5_D | 0.190 | 40 | 40 | 0.001 | 999 | Significant |
| RAIO_S3_A | DOLCE AGOGIA_S5_D | 0.100 | 40 | 40 | 0.001 | 999 | Significant |
| RAIO_S3_B | DOLCE AGOGIA_S5_D | 0.126 | 40 | 40 | 0.001 | 999 | Significant |
| RAIO_S3_C | DOLCE AGOGIA_S5_D | 0.125 | 40 | 40 | 0.001 | 999 | Significant |
| RAIO_S3_D | DOLCE AGOGIA_S5_D | 0.091 | 40 | 40 | 0.001 | 999 | Significant |
| BORGIONA_S4_A | DOLCE AGOGIA_S5_D | 0.172 | 40 | 40 | 0.001 | 999 | Significant |
| BORGIONA_S4_B | DOLCE AGOGIA_S5_D | 0.152 | 40 | 40 | 0.001 | 999 | Significant |
| BORGIONA_S4_C | DOLCE AGOGIA_S5_D | 0.121 | 40 | 40 | 0.001 | 999 | Significant |
| BORGIONA_S4_D | DOLCE AGOGIA_S5_D | 0.158 | 40 | 40 | 0.001 | 999 | Significant |
| **GENTILE GRANDE_S4_A** | DOLCE AGOGIA_S5_D | 0.089 | 40 | 40 | 0.001 | 999 | Significant |
| **GENTILE GRANDE_S4_B** | DOLCE AGOGIA_S5_D | 0.067 | 40 | 40 | 0.001 | 999 | Significant |
| **GENTILE GRANDE_S4_C** | DOLCE AGOGIA_S5_D | 0.083 | 40 | 40 | 0.001 | 999 | Significant |
| **GENTILE GRANDE_S4_D** | DOLCE AGOGIA_S5_D | 0.077 | 40 | 40 | 0.001 | 999 | Significant |
| DOLCE AGOGIA_S5_A | DOLCE AGOGIA_S5_D | 0.000 | 40 | 40 | 0.425 | 999 | NS |
| DOLCE AGOGIA_S5_B | DOLCE AGOGIA_S5_D | 0.000 | 40 | 40 | 0.438 | 999 | NS |
| DOLCE AGOGIA_S5_C | DOLCE AGOGIA_S5_D | 0.000 | 40 | 40 | 0.436 | 999 | NS |
| **MORAIOLO_S1_A** | **LECCINO_S5_A** | 0.257 | 40 | 40 | 0.001 | 999 | Significant |
| **MORAIOLO_S1_B** | **LECCINO_S5_A** | 0.238 | 40 | 40 | 0.001 | 999 | Significant |
| **MORAIOLO_S1_C** | **LECCINO_S5_A** | 0.254 | 40 | 40 | 0.001 | 999 | Significant |
| **MORAIOLO_S1_D** | **LECCINO_S5_A** | 0.253 | 40 | 40 | 0.001 | 999 | Significant |
| **SAN FELICE_S1_A** | **LECCINO_S5_A** | 0.184 | 40 | 40 | 0.001 | 999 | Significant |
| **SAN FELICE_S1_B** | **LECCINO_S5_A** | 0.144 | 40 | 40 | 0.001 | 999 | Significant |
| **SAN FELICE_S1_C** | **LECCINO_S5_A** | 0.191 | 40 | 40 | 0.001 | 999 | Significant |
| **SAN FELICE_S1_D** | **LECCINO_S5_A** | 0.196 | 40 | 40 | 0.001 | 999 | Significant |
| **MORAIOLO_S2_A** | **LECCINO_S5_A** | 0.175 | 40 | 40 | 0.001 | 999 | Significant |
| **MORAIOLO_S2_B** | **LECCINO_S5_A** | 0.170 | 40 | 40 | 0.001 | 999 | Significant |
| **MORAIOLO_S2_C** | **LECCINO_S5_A** | 0.186 | 40 | 40 | 0.001 | 999 | Significant |
| **MORAIOLO_S2_D** | **LECCINO_S5_A** | 0.185 | 40 | 40 | 0.001 | 999 | Significant |
| **MORAIOLO_S3_A** | **LECCINO_S5_A** | 0.317 | 40 | 40 | 0.001 | 999 | Significant |
| **MORAIOLO_S3_B** | **LECCINO_S5_A** | 0.299 | 40 | 40 | 0.001 | 999 | Significant |
| **MORAIOLO_S3_C** | **LECCINO_S5_A** | 0.313 | 40 | 40 | 0.001 | 999 | Significant |
| **MORAIOLO_S3_D** | **LECCINO_S5_A** | 0.310 | 40 | 40 | 0.001 | 999 | Significant |
| RAIO_S3_A | **LECCINO_S5_A** | 0.220 | 40 | 40 | 0.001 | 999 | Significant |
| RAIO_S3_B | **LECCINO_S5_A** | 0.266 | 40 | 40 | 0.001 | 999 | Significant |
| RAIO_S3_C | **LECCINO_S5_A** | 0.253 | 40 | 40 | 0.001 | 999 | Significant |
| RAIO_S3_D | **LECCINO_S5_A** | 0.229 | 40 | 40 | 0.001 | 999 | Significant |
| BORGIONA_S4_A | **LECCINO_S5_A** | 0.230 | 40 | 40 | 0.001 | 999 | Significant |
| BORGIONA_S4_B | **LECCINO_S5_A** | 0.221 | 40 | 40 | 0.001 | 999 | Significant |
| BORGIONA_S4_C | **LECCINO_S5_A** | 0.202 | 40 | 40 | 0.001 | 999 | Significant |
| BORGIONA_S4_D | **LECCINO_S5_A** | 0.241 | 40 | 40 | 0.001 | 999 | Significant |
| **GENTILE GRANDE_S4_A** | **LECCINO_S5_A** | 0.098 | 40 | 40 | 0.001 | 999 | Significant |
| **GENTILE GRANDE_S4_B** | **LECCINO_S5_A** | 0.120 | 40 | 40 | 0.001 | 999 | Significant |
| **GENTILE GRANDE_S4_C** | **LECCINO_S5_A** | 0.091 | 40 | 40 | 0.001 | 999 | Significant |
| **GENTILE GRANDE_S4_D** | **LECCINO_S5_A** | 0.116 | 40 | 40 | 0.001 | 999 | Significant |
| DOLCE AGOGIA_S5_A | **LECCINO_S5_A** | 0.158 | 40 | 40 | 0.001 | 999 | Significant |
| DOLCE AGOGIA_S5_B | **LECCINO_S5_A** | 0.144 | 40 | 40 | 0.001 | 999 | Significant |
| DOLCE AGOGIA_S5_C | **LECCINO_S5_A** | 0.147 | 40 | 40 | 0.001 | 999 | Significant |
| DOLCE AGOGIA_S5_D | **LECCINO_S5_A** | 0.147 | 40 | 40 | 0.001 | 999 | Significant |
| **MORAIOLO_S1_A** | **LECCINO_S5_B** | 0.225 | 40 | 40 | 0.001 | 999 | Significant |
| **MORAIOLO_S1_B** | **LECCINO_S5_B** | 0.203 | 40 | 40 | 0.001 | 999 | Significant |
| **MORAIOLO_S1_C** | **LECCINO_S5_B** | 0.223 | 40 | 40 | 0.001 | 999 | Significant |
| **MORAIOLO_S1_D** | **LECCINO_S5_B** | 0.220 | 40 | 40 | 0.001 | 999 | Significant |
| **SAN FELICE_S1_A** | **LECCINO_S5_B** | 0.159 | 40 | 40 | 0.001 | 999 | Significant |
| **SAN FELICE_S1_B** | **LECCINO_S5_B** | 0.132 | 40 | 40 | 0.001 | 999 | Significant |
| **SAN FELICE_S1_C** | **LECCINO_S5_B** | 0.168 | 40 | 40 | 0.001 | 999 | Significant |
| **SAN FELICE_S1_D** | **LECCINO_S5_B** | 0.182 | 40 | 40 | 0.001 | 999 | Significant |
| **MORAIOLO_S2_A** | **LECCINO_S5_B** | 0.173 | 40 | 40 | 0.001 | 999 | Significant |
| **MORAIOLO_S2_B** | **LECCINO_S5_B** | 0.161 | 40 | 40 | 0.001 | 999 | Significant |
| **MORAIOLO_S2_C** | **LECCINO_S5_B** | 0.181 | 40 | 40 | 0.001 | 999 | Significant |
| **MORAIOLO_S2_D** | **LECCINO_S5_B** | 0.183 | 40 | 40 | 0.001 | 999 | Significant |
| **MORAIOLO_S3_A** | **LECCINO_S5_B** | 0.325 | 40 | 40 | 0.001 | 999 | Significant |
| **MORAIOLO_S3_B** | **LECCINO_S5_B** | 0.306 | 40 | 40 | 0.001 | 999 | Significant |
| **MORAIOLO_S3_C** | **LECCINO_S5_B** | 0.325 | 40 | 40 | 0.001 | 999 | Significant |
| **MORAIOLO_S3_D** | **LECCINO_S5_B** | 0.320 | 40 | 40 | 0.001 | 999 | Significant |
| RAIO_S3_A | **LECCINO_S5_B** | 0.218 | 40 | 40 | 0.001 | 999 | Significant |
| RAIO_S3_B | **LECCINO_S5_B** | 0.269 | 40 | 40 | 0.001 | 999 | Significant |
| RAIO_S3_C | **LECCINO_S5_B** | 0.249 | 40 | 40 | 0.001 | 999 | Significant |
| RAIO_S3_D | **LECCINO_S5_B** | 0.225 | 40 | 40 | 0.001 | 999 | Significant |
| BORGIONA_S4_A | **LECCINO_S5_B** | 0.235 | 40 | 40 | 0.001 | 999 | Significant |
| BORGIONA_S4_B | **LECCINO_S5_B** | 0.220 | 40 | 40 | 0.001 | 999 | Significant |
| BORGIONA_S4_C | **LECCINO_S5_B** | 0.207 | 40 | 40 | 0.001 | 999 | Significant |
| BORGIONA_S4_D | **LECCINO_S5_B** | 0.240 | 40 | 40 | 0.001 | 999 | Significant |
| **GENTILE GRANDE_S4_A** | **LECCINO_S5_B** | 0.129 | 40 | 40 | 0.001 | 999 | Significant |
| **GENTILE GRANDE_S4_B** | **LECCINO_S5_B** | 0.142 | 40 | 40 | 0.001 | 999 | Significant |
| **GENTILE GRANDE_S4_C** | **LECCINO_S5_B** | 0.125 | 40 | 40 | 0.001 | 999 | Significant |
| **GENTILE GRANDE_S4_D** | **LECCINO_S5_B** | 0.148 | 40 | 40 | 0.001 | 999 | Significant |
| DOLCE AGOGIA_S5_A | **LECCINO_S5_B** | 0.149 | 40 | 40 | 0.001 | 999 | Significant |
| DOLCE AGOGIA_S5_B | **LECCINO_S5_B** | 0.148 | 40 | 40 | 0.001 | 999 | Significant |
| DOLCE AGOGIA_S5_C | **LECCINO_S5_B** | 0.146 | 40 | 40 | 0.001 | 999 | Significant |
| DOLCE AGOGIA_S5_D | **LECCINO_S5_B** | 0.140 | 40 | 40 | 0.001 | 999 | Significant |
| **LECCINO_S5_A** | **LECCINO_S5_B** | 0.013 | 40 | 40 | 0.077 | 999 | NS |
| **MORAIOLO_S1_A** | **LECCINO_S5_C** | 0.226 | 40 | 40 | 0.001 | 999 | Significant |
| **MORAIOLO_S1_B** | **LECCINO_S5_C** | 0.204 | 40 | 40 | 0.001 | 999 | Significant |
| **MORAIOLO_S1_C** | **LECCINO_S5_C** | 0.222 | 40 | 40 | 0.001 | 999 | Significant |
| **MORAIOLO_S1_D** | **LECCINO_S5_C** | 0.220 | 40 | 40 | 0.001 | 999 | Significant |
| **SAN FELICE_S1_A** | **LECCINO_S5_C** | 0.154 | 40 | 40 | 0.001 | 999 | Significant |
| **SAN FELICE_S1_B** | **LECCINO_S5_C** | 0.124 | 40 | 40 | 0.001 | 999 | Significant |
| **SAN FELICE_S1_C** | **LECCINO_S5_C** | 0.163 | 40 | 40 | 0.001 | 999 | Significant |
| **SAN FELICE_S1_D** | **LECCINO_S5_C** | 0.174 | 40 | 40 | 0.001 | 999 | Significant |
| **MORAIOLO_S2_A** | **LECCINO_S5_C** | 0.163 | 40 | 40 | 0.001 | 999 | Significant |
| **MORAIOLO_S2_B** | **LECCINO_S5_C** | 0.156 | 40 | 40 | 0.001 | 999 | Significant |
| **MORAIOLO_S2_C** | **LECCINO_S5_C** | 0.172 | 40 | 40 | 0.001 | 999 | Significant |
| **MORAIOLO_S2_D** | **LECCINO_S5_C** | 0.172 | 40 | 40 | 0.001 | 999 | Significant |
| **MORAIOLO_S3_A** | **LECCINO_S5_C** | 0.302 | 40 | 40 | 0.001 | 999 | Significant |
| **MORAIOLO_S3_B** | **LECCINO_S5_C** | 0.285 | 40 | 40 | 0.001 | 999 | Significant |
| **MORAIOLO_S3_C** | **LECCINO_S5_C** | 0.301 | 40 | 40 | 0.001 | 999 | Significant |
| **MORAIOLO_S3_D** | **LECCINO_S5_C** | 0.296 | 40 | 40 | 0.001 | 999 | Significant |
| RAIO_S3_A | **LECCINO_S5_C** | 0.204 | 40 | 40 | 0.001 | 999 | Significant |
| RAIO_S3_B | **LECCINO_S5_C** | 0.251 | 40 | 40 | 0.001 | 999 | Significant |
| RAIO_S3_C | **LECCINO_S5_C** | 0.234 | 40 | 40 | 0.001 | 999 | Significant |
| RAIO_S3_D | **LECCINO_S5_C** | 0.207 | 40 | 40 | 0.001 | 999 | Significant |
| BORGIONA_S4_A | **LECCINO_S5_C** | 0.222 | 40 | 40 | 0.001 | 999 | Significant |
| BORGIONA_S4_B | **LECCINO_S5_C** | 0.207 | 40 | 40 | 0.001 | 999 | Significant |
| BORGIONA_S4_C | **LECCINO_S5_C** | 0.196 | 40 | 40 | 0.001 | 999 | Significant |
| BORGIONA_S4_D | **LECCINO_S5_C** | 0.225 | 40 | 40 | 0.001 | 999 | Significant |
| **GENTILE GRANDE_S4_A** | **LECCINO_S5_C** | 0.100 | 40 | 40 | 0.001 | 999 | Significant |
| **GENTILE GRANDE_S4_B** | **LECCINO_S5_C** | 0.113 | 40 | 40 | 0.001 | 999 | Significant |
| **GENTILE GRANDE_S4_C** | **LECCINO_S5_C** | 0.093 | 40 | 40 | 0.001 | 999 | Significant |
| **GENTILE GRANDE_S4_D** | **LECCINO_S5_C** | 0.119 | 40 | 40 | 0.001 | 999 | Significant |
| DOLCE AGOGIA_S5_A | **LECCINO_S5_C** | 0.135 | 40 | 40 | 0.001 | 999 | Significant |
| DOLCE AGOGIA_S5_B | **LECCINO_S5_C** | 0.129 | 40 | 40 | 0.001 | 999 | Significant |
| DOLCE AGOGIA_S5_C | **LECCINO_S5_C** | 0.130 | 40 | 40 | 0.001 | 999 | Significant |
| DOLCE AGOGIA_S5_D | **LECCINO_S5_C** | 0.124 | 40 | 40 | 0.001 | 999 | Significant |
| **LECCINO_S5_A** | **LECCINO_S5_C** | 0.000 | 40 | 40 | 0.426 | 999 | NS |
| **LECCINO_S5_B** | **LECCINO_S5_C** | 0.000 | 40 | 40 | 0.421 | 999 | NS |
| **MORAIOLO_S1_A** | **LECCINO_S5_D** | 0.262 | 40 | 40 | 0.001 | 999 | Significant |
| **MORAIOLO_S1_B** | **LECCINO_S5_D** | 0.241 | 40 | 40 | 0.001 | 999 | Significant |
| **MORAIOLO_S1_C** | **LECCINO_S5_D** | 0.259 | 40 | 40 | 0.001 | 999 | Significant |
| **MORAIOLO_S1_D** | **LECCINO_S5_D** | 0.257 | 40 | 40 | 0.001 | 999 | Significant |
| **SAN FELICE_S1_A** | **LECCINO_S5_D** | 0.189 | 40 | 40 | 0.001 | 999 | Significant |
| **SAN FELICE_S1_B** | **LECCINO_S5_D** | 0.153 | 40 | 40 | 0.001 | 999 | Significant |
| **SAN FELICE_S1_C** | **LECCINO_S5_D** | 0.200 | 40 | 40 | 0.001 | 999 | Significant |
| **SAN FELICE_S1_D** | **LECCINO_S5_D** | 0.205 | 40 | 40 | 0.001 | 999 | Significant |
| **MORAIOLO_S2_A** | **LECCINO_S5_D** | 0.184 | 40 | 40 | 0.001 | 999 | Significant |
| **MORAIOLO_S2_B** | **LECCINO_S5_D** | 0.176 | 40 | 40 | 0.001 | 999 | Significant |
| **MORAIOLO_S2_C** | **LECCINO_S5_D** | 0.190 | 40 | 40 | 0.001 | 999 | Significant |
| **MORAIOLO_S2_D** | **LECCINO_S5_D** | 0.189 | 40 | 40 | 0.001 | 999 | Significant |
| **MORAIOLO_S3_A** | **LECCINO_S5_D** | 0.330 | 40 | 40 | 0.001 | 999 | Significant |
| **MORAIOLO_S3_B** | **LECCINO_S5_D** | 0.311 | 40 | 40 | 0.001 | 999 | Significant |
| **MORAIOLO_S3_C** | **LECCINO_S5_D** | 0.326 | 40 | 40 | 0.001 | 999 | Significant |
| **MORAIOLO_S3_D** | **LECCINO_S5_D** | 0.323 | 40 | 40 | 0.001 | 999 | Significant |
| RAIO_S3_A | **LECCINO_S5_D** | 0.230 | 40 | 40 | 0.001 | 999 | Significant |
| RAIO_S3_B | **LECCINO_S5_D** | 0.276 | 40 | 40 | 0.001 | 999 | Significant |
| RAIO_S3_C | **LECCINO_S5_D** | 0.262 | 40 | 40 | 0.001 | 999 | Significant |
| RAIO_S3_D | **LECCINO_S5_D** | 0.235 | 40 | 40 | 0.001 | 999 | Significant |
| BORGIONA_S4_A | **LECCINO_S5_D** | 0.255 | 40 | 40 | 0.001 | 999 | Significant |
| BORGIONA_S4_B | **LECCINO_S5_D** | 0.239 | 40 | 40 | 0.001 | 999 | Significant |
| BORGIONA_S4_C | **LECCINO_S5_D** | 0.220 | 40 | 40 | 0.001 | 999 | Significant |
| BORGIONA_S4_D | **LECCINO_S5_D** | 0.260 | 40 | 40 | 0.001 | 999 | Significant |
| **GENTILE GRANDE_S4_A** | **LECCINO_S5_D** | 0.107 | 40 | 40 | 0.001 | 999 | Significant |
| **GENTILE GRANDE_S4_B** | **LECCINO_S5_D** | 0.131 | 40 | 40 | 0.001 | 999 | Significant |
| **GENTILE GRANDE_S4_C** | **LECCINO_S5_D** | 0.108 | 40 | 40 | 0.001 | 999 | Significant |
| **GENTILE GRANDE_S4_D** | **LECCINO_S5_D** | 0.133 | 40 | 40 | 0.001 | 999 | Significant |
| DOLCE AGOGIA_S5_A | **LECCINO_S5_D** | 0.158 | 40 | 40 | 0.001 | 999 | Significant |
| DOLCE AGOGIA_S5_B | **LECCINO_S5_D** | 0.150 | 40 | 40 | 0.001 | 999 | Significant |
| DOLCE AGOGIA_S5_C | **LECCINO_S5_D** | 0.151 | 40 | 40 | 0.001 | 999 | Significant |
| DOLCE AGOGIA_S5_D | **LECCINO_S5_D** | 0.148 | 40 | 40 | 0.001 | 999 | Significant |
| **LECCINO_S5_A** | **LECCINO_S5_D** | 0.000 | 40 | 40 | 0.407 | 999 | NS |
| **LECCINO_S5_B** | **LECCINO_S5_D** | 0.000 | 40 | 40 | 0.429 | 999 | NS |
| **LECCINO_S5_C** | **LECCINO_S5_D** | 0.000 | 40 | 40 | 0.433 | 999 | NS |
